# An antibody-free sample pretreatment method for osteopontin combined with MALDI-TOF MS/MS analysis

**DOI:** 10.1371/journal.pone.0213405

**Published:** 2019-03-07

**Authors:** Yuye Zhou, Joakim Romson, Åsa Emmer

**Affiliations:** KTH Royal Institute of Technology, School of Engineering Sciences in Chemistry, Biotechnology and Health, Department of Chemistry, Division of Applied Physical Chemistry, Analytical Chemistry, Stockholm, Sweden; University of Missouri Columbia, UNITED STATES

## Abstract

Osteopontin is an osteoblast-secreted protein with an aspartic acid-rich, highly phosphorylated, and glycosylated structure. Osteopontin can easily bind to integrins, tumor cells, extracellular matrix and calcium, and is related to bone diseases, various cancers, inflammation *etc*. Here, DEAE-Cibacron blue 3GA was used to extract recombinant osteopontin from human plasma, and to deplete abundant plasma proteins with an antibody-free method. Using selected buffer systems, osteopontin and human serum albumin could be bound to DEAE-Cibacron blue 3GA, while immunoglobulin G was excluded. The bound osteopontin could then be separated from albumin by using different sequential elution buffers. By this method, 1 μg/mL recombinant osteopontin could be separated from the major part of the most abundant proteins in human plasma. After trypsin digestion, the extracted osteopontin could be successfully detected and identified by MALDI-TOF MS/MS using the *m/z* 1854.898 peptide and its fragments.

## Introduction

Human osteopontin (OPN) is a multifunctional protein secreted by a variety of cells such as activated macrophages, leukocytes, activated T lymphocytes and osteoblastic cells, and it is highly implicated in bone mineralization, inflammation, autoimmune diseases, and some types of cancer [1–5]. OPN is expressed in many body fluids, such as plasma, urine, and cerebrospinal fluid [6–8], and is subject to extensive post-translational modifications such as glycosylations and phosphorylations [9]. Phosphorylation and the richness of aspartic acid and glutamic acid cause OPN to have affinity to hydroxyapatatite (a mineral present in bone and teeth), and to calcium ions [8, 10]. It has also been found that some domains within OPN contribute to cell binding [11]. For example, the arginine-glycine-aspartate (RGD) domain can interact with cell surface αvβ3 intergrin. This interaction facilitates the attachment of osteoclasts to the bone matrix, where OPN is one of the most abundant proteins. OPN serves as a bone-bridge and provides links between bone regeneration and the immune system [5, 12]. OPN is also implicated as a ligand in the interaction with CD44 surface receptors that regulate cell adhesion and movement, and also serves to activate both healthy immune cells and neoplastic cells [1, 13, 14]. By interacting with cell surface integrins and CD44 variants, OPN stimulates cell adhesion, migration or survival, and regulate multiple tumor promoting mechanisms [3, 15]. The 2.5 to 97.5 percentile of OPN plasma concentration in healthy subjects is <7.8 to 87 ng/mL, with a median of 51 ng/mL [16], but the levels are elevated in many diseases such as multiple sclerosis [17], and several types of cancer [18–24]. Even higher levels than 1 μg/mL OPN in human plasma have been reported in many individual cases, including coronary artery disease [25], heart failure [26], prostate carcinoma [18], hepatocellular carcinoma [21], breast cancer [19, 22], and cervix cancer [27]. Thus, OPN can be used as a biomarker by comparing the levels found for patients potentially subjected to these diseases with healthy controls.

Protein measurements in human plasma or serum are often dependent on the availability of immunoassays, particularly commercial enzyme-linked immunosorbent assay (ELISA) systems [28]. However, several factors may cause variability and inaccuracy of the immunoassay measurements, such as the cross-reactivity of the antibodies used, post-translational modifications in measured proteins, and interferences due to autoantibodies and anti-reagent antibodies [29]. The analysis of human OPN has also been shown to be influenced by the choice of commercial ELISA kit, possibly due to different isoforms and post translational modifications of OPN, and recognition epitopes of the antibody [27, 30–32]. Recently, tremendous progress has been made in the application of mass spectrometry (MS) for quantification of proteins by measuring peptides originating from proteolytic digestion. The identification and quantification of the low abundant protein OPN in human plasma was enabled by tandem MS in combination with immunoaffinity capture methods [33–35]. MALDI TOF/TOF tandem MS has also been used in previous work for OPN characterization [36]. Nevertheless, all methods mentioned above require the utilization of affinity methods, demanding well characterized antibodies with high specificity for OPN in plasma, which in turn implies high costs. Due to the high abundance of proteins such as human serum albumin (HSA) and immunoglobulins (Igs) in human plasma, detection of low concentrations of OPN will be difficult to achieve without enrichment using antibodies, though. Thus, the development of a fast and antibody-free method for isolation, detection and identification of human OPN is important and still a challenge.

Protein A is a Staphylococcal protein capable of precipitating human Igs [37–39], and by immobilizing it on sepharose an affinity column for Igs is obtained [40]. An affinity column utilizing the coupling of Cibacron blue F-3-GA (CB) to Sepharose, on the other hand, enables removal of 98% of HSA [41]. Further resolution of proteins in addition to CB treatment could be achieved by using weak ion exchange chromatography based on diethylaminoethyl (DEAE) cellulose [41], developed by Hoffpauir et al. [42]. DEAE-Affi-Gel Blue, the combination of ion-exchange and pseudo-ligand-affinity materials, has also been reported in human serum protein chromatography [43].

In the present work, protein A−Agarose and DEAE−Cibacron Blue 3GA−Agarose (DEAE-CB) were investigated for OPN enrichment. A novel antibody-free strategy based on DEAE-CB separation and MALDI tandem MS characterization was developed to extract recombinant human OPN (rhOPN) from human plasma in μL sized samples. The DEAE-CB extraction method was used in combination with sequential buffer systems designed to extract rhOPN from human plasma in a selective way. The detection and identification of extracted rhOPN was focused on a fingerprint peptide obtained through trypsin digestion.

## Materials and methods

### Materials

Acetonitrile (ACN), trifluoroacetic acid (TFA), albumin (A3059, from bovine serum, BSA), Immunoglobulin G (I5506, IgG from bovine serum), Protein A–Agarose (P1406), DEAE−Cibacron Blue 3GA−Agarose (D0407, DEAE-CB), Millipore Ziptip (C18, 10 μL, Z720070-96EA), trypsin (T1426, from bovine pancreas), recombinant human osteopontin (rhOPN, SRP3131), human plasma (P9523), and all the salts were obtained from Sigma-Aldrich (Stockholm, Sweden). All water (MQ H_2_O) used was purified in a Millipore Synergy 185 (Bedford, MA, USA) to a resistivity of 18.2 MΩ·cm at 25°C. Human plasma was stored in -80°C, and rhOPN was reconstituted in MQ H_2_O to 1 mg/mL and then stored in -20°C. Pierce C18 Tips, 100 μL bed, were from Thermo Fisher Scientific (Rockford, USA). Eppendorf LoBind microcentrifuge tubes (Lobind tube) from Sigma-Aldrich were used for all protein and peptide samples. Eppendorf ThermoMixer C (Hamburg, Germany) was used for both protein isolation (at 1200 rpm) and trypsin digestion (at 1500 rpm). MALDI matrix 2,5-dihydroxybenzoic acid (DHB), α-cyano-4-hydroxycinnamic acid (HCCA), Peptide Calibration Standard, Protein Calibration Standard I and II, MALDI plates ground steel (GS) and MTP AnchorChip 384 BC (AC) were purchased from Bruker Daltonics (Bremen, Germany). Alkyl ketene dimer (AKD) was from Eka Chemicals (Bohus, Sweden) and was a gift from Cellutech AB (Stockholm, Sweden). Ethanol (96%) was from VWR (Stockholm, Sweden).

### rhOPN extraction from protein mixture and human plasma

rhOPN (10 μL, 200 μg /mL) or a protein mixture of 5 μL BSA (40 mg/mL), 5 μL bovine IgG (10 mg/mL) and 5 μL rhOPN (200 μg /mL) were used for isolation of rhOPN using 10 mg Protein A or 20 μL DEAE-CB. Protein A was swollen in 50 μL binding buffer (10 mM Tris-HCl pH 8.0) for 30 min before use. Both affinity materials were conditioned with 100 μL binding buffer for 5 min in 1.5 mL LoBind tubes, and the supernatant liquids were discarded after centrifugation using Biofuge Pico microcentrifuge (Langenselbold, Germany), for all steps the centrifugation was carried out at 2400 xg for 3 min. The rhOPN or the protein mixture was diluted with 15 μL binding buffer, and was mixed with the affinity material for 30 min (all mixing steps were carried out in ThermoMixer C at 1200 rpm). The supernatant after centrifugation was collected as “Non-binding fraction”. The affinity material was then washed with 10 affinity material volumes (MV) of binding buffer for 30 min and the supernatant liquid was collected as “Washing fraction”. The bound proteins were eluted with 10 MV of 10 mM Tris-HCl + 1.5 M NaCl pH 8.0 (Tris-NaCl pH 8.0) and 10 MV of 0.2 M NaH_2_PO_4_ + 0.2 M Glycine pH 4.4 buffer (PB-Gly pH 4.4) for 30 min respectively, the supernatant liquids after centrifugation were collected as “Tris-NaCl pH 8.0 Elution” and “PB-Gly pH 4.4 Elution”. All steps were carried out at room temperature.

**Fig 1:**
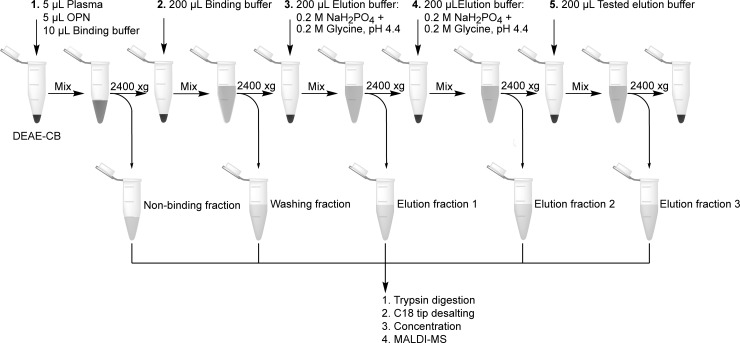
Schematics of the enrichment method for rhOPN in plasma samples (sample and buffer volumes were doubled for 1 μg/ml rhOPN in plasma samples).

Plasma samples, 5 μL, were spiked with 5 μL rhOPN solutions of different concentrations (2 μg/mL to 1 mg/mL) and diluted with 10 μL of the different assessed binding buffers respectively before mixing with preconditioned 20 μL DEAE-CB for 30 min. When 1 μg/ml rhOPN in plasma was investigated all sample and buffer volumes were doubled. The binding and washing steps were the same as used for protein mixture samples. The remaining proteins attached to DEAE-CB were eluted with 10 MV of PB-Gly pH 4.4 twice, the centrifuged supernatant liquids were collected as “Elution fraction 1” and “Elution fraction 2” respectively. Finally, the proteins still attached to the DEAE-CB were eluted with 10 MV of the assessed elution buffers and collected as “Elution fraction 3”. When 1 mg/mL rhOPN in human plasma was investigated, all fractions were collected separately and divided into two aliquots respectively. One aliquot was analyzed regarding intact protein and the other was analyzed after trypsin digestion. Tris-HCl and NaH_2_PO_4_ buffers with different pH values (pH 4.0, 6.0, 8.0) and concentrations (10 mM and 100 mM) were studied for binding. 10 mM Tris-HCl pH 8.0 and PB-Gly pH 4.4 buffers with NaCl concentration 0.1 M, 0.2 M, 0.3 M, 0.4 M, 0.5 M and 1.5 M were applied sequentially after the first elution with PB-Gly pH 4.4. In total, six elution fractions were collected for trypsin digestion. Buffers for the third elution fraction were evaluated at pH 4.4 and 8.0 with 1 μg/mL rhOPN in plasma. The procedures for binding, washing and elution on plasma samples with DEAE-CB are shown in Fig 1 and all buffers investigated are listed in Table 1.

**Table 1:** Binding and elution buffers evaluated with protein mixture samples and rhOPN in human plasma samples.

**Sample information**	**Binding buffer**	**Elution buffer 1**	**Elution buffer 2**	**Elution buffer 3**
Protein mixture	10 mM Tris-HCl pH 8.0	10 mM Tris-HCl + 1.5 M NaCl pH 8.0 (Tris-NaCl pH 8.0)	0.2 M NaH_2_PO_4_ + 0.2 M Glycine pH 4.4 (PB-Gly pH 4.4)	-
0.1–1 mg/mL rhOPN in plasma	10 mM Tris-HCl pH 8.0	PB-Gly pH 4.4	PB-Gly pH 4.4	Tris-NaCl pH 8.0
**Sample information** ***Elution salt conc*. *evaluation***	**Binding buffer**	**Elution buffer 1**	**Elution order**	
20 μg/mL rhOPN in plasma with Tris-HCl	10 mM Tris-HCl pH 4.0	PB-Gly pH 4.4	Eluted by following order (six fractions were collected): 10 mM Tris-HCl + 0.1 M NaCl pH 8.0 10 mM Tris-HCl + 0.2 M NaCl pH 8.0 10 mM Tris-HCl + 0.3 M NaCl pH 8.0 10 mM Tris-HCl + 0.4 M NaCl pH 8.0 10 mM Tris-HCl + 0.5 M NaCl pH 8.0 10 mM Tris-HCl + 1.5 M NaCl pH 8.0	
20 μg/mL rhOPN in plasma with NaH_2_PO_4_	10 mM NaH_2_PO_4_ pH 4.0	PB-Gly pH 4.4	Eluted by following order (six fractions were collected): 0.2 M NaH_2_PO_4_ + 0.2 M Glycine + 0.1 M NaCl pH 4.4 0.2 M NaH_2_PO_4_ + 0.2 M Glycine + 0.2 M NaCl pH 4.4 0.2 M NaH_2_PO_4_ + 0.2 M Glycine + 0.3 M NaCl pH 4.4 0.2 M NaH_2_PO_4_ + 0.2 M Glycine + 0.4 M NaCl pH 4.4 0.2 M NaH_2_PO_4_ + 0.2 M Glycine + 0.5 M NaCl pH 4.4 0.2 M NaH_2_PO_4_ + 0.2 M Glycine + 1.5 M NaCl pH 4.4	
**Sample information** ***Binding and elution buffers evaluation***	**Binding buffer**	**Elution buffer 1**	**Elution buffer 2**	**Elution buffer 3**
20 μg/mL rhOPN in plasma	10 mM Tris-HCl pH 4.0 or pH 8.0	PB-Gly pH 4.4	PB-Gly pH 4.4	Tris-NaCl pH 8.0
	10 mM NaH_2_PO_4_ pH 4.0 or pH 8.0	PB-Gly pH 4.4	PB-Gly pH 4.4	Tris-NaCl pH 8.0
2 μg/ml—4 μg/ml rhOPN in plasma	10 mM NaH_2_PO_4_ pH 4.0 or pH 6.0 or pH 8.0	PB-Gly pH 4.4	PB-Gly pH 4.4	0.2 M NaH_2_PO_4_ + 0.2 M Glycine + 0.3 M NaCl pH 4.4 or Tris-NaCl pH 8.0
	100 mM NaH_2_PO_4_ pH 4.0 or pH 6.0 or pH 8.0	PB-Gly pH 4.4	PB-Gly pH 4.4	0.2 M NaH_2_PO_4_ + 0.2 M Glycine + 0.3 M NaCl pH 4.4 or Tris-NaCl pH 8.0
**Sample information** ***Elution pH evaluation***	**Binding buffer**	**Elution buffer 1**	**Elution buffer 2**	**Elution buffer 3**
1 μg/ml rhOPN in plasma	100 mM NaH_2_PO_4_ pH 4.0	PB-Gly pH 4.4	PB-Gly pH 4.4	0.2 M NaH_2_PO_4_ + 0.2 M Glycine + 0.3 M NaCl pH 4.4
	100 mM NaH_2_PO_4_ pH 4.0	PB-Gly pH 4.4	PB-Gly pH 4.4	0.2 M NaH_2_PO_4_ + 0.2 M Glycine + 0.3 M NaCl pH 8.0

NaH_2_PO_4_ concentrations are 10 mM or 100 mM in binding buffers, and 0.2 M in elution buffers.

### Trypsin digestion

rhOPN reference samples (100 ng/mL to 1 mg/mL in MQ H_2_O) were mixed with trypsin and NH_4_HCO_3_, pH 8.0, in LoBind tubes for digestion (concentrations and volumes of rhOPN, trypsin and salt are listed in S1 Table). DEAE-CB collected fractions, for both protein mixture and plasma samples, were adjusted to a pH around 7.5 with 1 M NaOH, and after that 1 μg trypsin was added to each fraction for digestion. For some of 1–2 μg/mL rhOPN in plasma samples, 0.25 or 0.5 μg trypsin was added. Denaturation was carried out before digestion on some of the DEAE-CB collected samples (2–4 μg/mL rhOPN in plasma) at 95°C for 10 min for comparison. All digestions were carried out in ThermoMixer C at 37°C for 17 h, and terminated at 95°C for 3 min.

### Dephosphorylation

Dephosphorylation was carried out by incubating 0.25 unit or 1 unit of alkaline phosphatase (P0114, Sigma-Aldrich, Stockholm, Sweden) with 10 μL of 100 μg/mL rhOPN in 5 mM Tris pH 7.9, 10 mM NaCl, 1 mM MgCl_2_, and 0.1 mM dithiothreitol for 30 min at 30°C, according to the manufacturer’s protocol. Dephosphorylation studies were carried out both before and after trypsin digestion, the molar ratio between rhOPN and trypsin was 2:1. All dephosphorylated samples were desalted before MALDI-MS analysis.

### Desalting and concentration

Millipore Ziptip was used to desalt 10 μL digested rhOPN reference solution (100–200 ng/mL) according to the manufacturer’s protocol (Merck Millipore Ltd, Cork, IRL, PR 02358, Rev A 02/07). Finally, the desalted peptides were eluted by 3 μL 50:50 (v/v) ACN: 0.1% TFA in H_2_O (TA 50). No desalting or concentration step was needed for 400 ng/mL—1 mg/mL rhOPN digested samples before MALDI-MS analysis. DEAE-CB collected and digested fractions were acidified by 1 μL TFA before desalting. Pierce C18 Tips were wetted 4 times with 100 μL 50% ACN in H_2_O, and conditioned 4 times with 100 μL 0.1% TFA in H_2_O. Sample fractions were aspirated and dispensed 10 times through the C18 bed. After that, the C18 bed was washed 3 times with 100 μL 5% ACN, 0.1% TFA in H_2_O. Then, the proteins or peptides were eluted by 50 μL of TA 50 and dried using Eppendorf concentrator 5301 (Hamburg, Germany). The dried samples were then dissolved in 2.5 μL of 30:70 (v/v) ACN: 0.1% TFA in H_2_O (TA 30) for MALDI-MS analysis. For all dephosphorylated samples, Pierce C18 Tips eluted fractions were concentrated to 20 μL.

### MALDI-TOF MS and MALDI-TOF MS/MS

MALDI AC plates and AKD structured plates were used for sample deposition. The AKD-plates with 600 μm anchors were manufactured as earlier described [44] with some changes. No dip-coating step was performed, instead the plate was covered with ca 50 layers of a finer grain mist (until the steel plate was not visible beneath the AKD layer) using aircap 1 and tip 1 on the Paasche H-model airbrush. 0.5 μL rhOPN intact standard protein solutions or trypsin digests were applied on a MALDI AC plate, while 0.5 μL DEAE-CB enriched proteins or digests were applied on an AKD structured plate, followed by an additional application of 0.5 μL of matrix after the sample droplet had dried. The DHB matrix used in the work was 20 mg/mL in TA 30, HCCA was evaluated at 2.8 mg/mL or saturated in TA 30. DHB was always used when analyzing rhOPN standard solutions in MS mode, and rhOPN (2 μg/mL– 1 mg/mL) in plasma samples in MS and MS/MS mode. HCCA was investigated for rhOPN (1 μg/mL– 4 μg/mL) in plasma samples in both modes. After crystallization, analysis was conducted on an UltrafleXtreme MALDI TOF/TOF Mass Spectrometer (Bruker Daltonics, Bremen, Germany) equipped with a SmartbeamII laser (355 nm, UV) in positive mode. External calibrations using protein or peptide calibration standards were performed every day before analyzing intact proteins in MS or daughter ions in MS/MS mode. Internal calibration was performed for trypsin digested samples using trypsin autolysis products. Intact protein samples were analyzed using linear mode, while peptides were analyzed using reflector mode. The laser was always adjusted to 80% of total intensity in MS mode and 90% in MS/MS mode. Each protein or peptide sample spot was analyzed with a sum of 5000 shots in MS mode, and 10000 shots in MS/MS mode. Mass spectra were recorded with flexControl and analyzed with FlexAnalysis Version 3.4 (Bruker Daltonics).

### OPN identification

The proteomics software tools (Bruker Biotools) were used for identification of proteins based on *m/z* data of digested peptides and fragmentation of selected peptides. The selected spectra were submitted to Mascot for search in the SwissProt database for MS ions or MS/MS ions. The species for data search was set as human, there was no amino acid modification included in the search. For MS search, the mass tolerance was 0.5 Da. In MS/MS search, the mass tolerance for the parent ions was 50 ppm and 1 Da for daughter ions.

## Results and discussion

### Trypsin digestion of rhOPN reference samples

**Fig 2:**
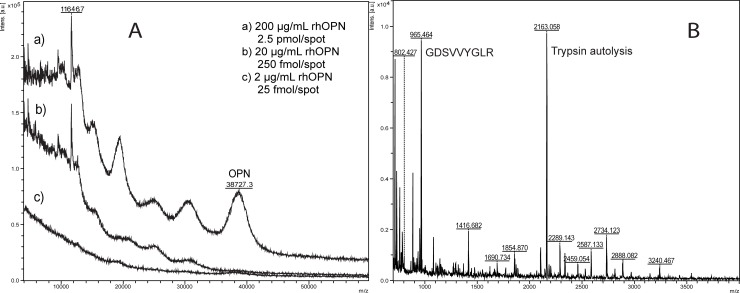
MALDI-MS of rhOPN. (A) Intact rhOPN. (B) Trypsin digest of 100 ng/mL rhOPN.

The intact rhOPN had a MW of 38.7 kDa with a detection limit of 250 fmol/spot (20 μg/mL) for the intact protein as determined with MALDI-TOF MS (Fig 2A). The detection and identification limit was 4.3 fmol/spot (100 ng/ml) for the digested peptides in MS mode using DHB as matrix. The digested peptides could be successfully identified as human OPN using Mascot data search using MS ions (Fig 2B, S1 Fig). The digested peptides which could be detected with MALDI-TOF MS are bold in the sequence shown below, and the sequence of the main peak *m/z* 965.505 (GDSVVYGLR) is underlined.

IPVKQADSGS 10 SEEKQLYNKY 20 PDAVATWLNP 30 DPSQK**QNLLA** 40 **PQNAVSSEET** 50 **NDFKQETLPS** 60 **K**SNESHDHMD 70 DMDDEDDDDH 80 VDSQDSIDSN 90 DSDDVDDTDD 100 SHQSDESHHS 110 DESDELVTDF 120 PTDLPATEVF 130 TPVVPTVDTY 140 DGR**GDSVVYG** 150 **LR**SKSKKFRR 160 PDIQYPDATD 170 EDITSHMESE 180 ELNGAYK**AIP** 190 **VAQDLNAPSD** 200 **WDSRGKDSYE** 210 **TSQLDDQSAE** 220 **THSHKQSR**LY 230 KR**KANDESNE** 240 **HSDVIDSQEL** 250 **SKVSR**EFHSH 260 EFHSHEDMLV 270 VDPK**SKEEDK** 280 **HLKFRISHEL** 290 **DSASSEVN** 298.

**Fig 3:**
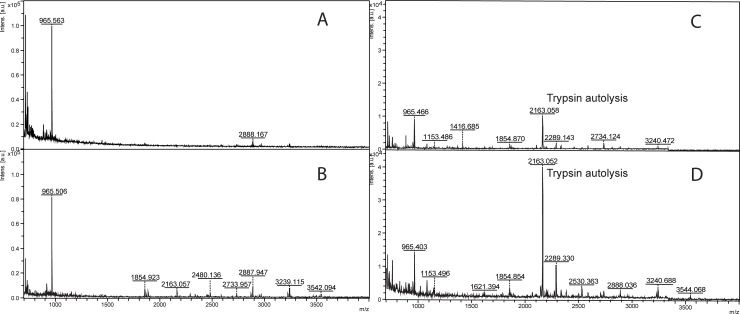
MALDI-MS of rhOPN trypsin digests with different molar ratio of trypsin to rhOPN. (A) 4 μg/mL rhOPN, n_trypsin_: n_rhOPN_ = 1:25, (B) 4 μg/mL rhOPN, n_trypsin_: n_rhOPN_ = 1:5, (C) 100 ng/mL rhOPN, n_trypsin_: n_rhOPN_ = 5:1, (D) 100 ng/mL rhOPN, n_trypsin_: n_rhOPN_ = 10:1.

When studying rhOPN trypsin digestion, it was found that the molar ratio of trypsin to rhOPN could affect the digestion and the resulting number of peptides detected. When changing the ratio of n_trypsin_: n_rhOPN_ from 1:25 to 1:5 for the digestion of 4 μg/mL rhOPN, more peptides could be detected and thus a higher score from Biotools (S2 Fig) could be obtained due to the higher sequence coverage (Fig 3A and 3B). A higher efficiency of the digestion could be accomplished when using a higher ratio of n_trypsin_: n_rhOPN_. This effect of increasing the ratio of n_trypsin_: n_rhOPN_ could also be seen for lower concentrations of rhOPN. For example, more peptides could be detected at the ratio 5:1 compared to 1:1 for 200 ng/mL rhOPN (S3 Fig). Moreover, at least a ratio of 5:1 was needed for the digestion of 100 ng/mL rhOPN (Fig 3C). There is no significant difference in the number of peaks and peak intensities for 100 ng/mL rhOPN when compairing the ratios of 10:1 and 5:1. However, the intensities for the trypsin autolysis peaks such as *m/z* 2163 were increased when the n_trypsin_: n_rhOPN_ ratio was raised to 10:1 (Fig 3D). Therefore, the n_trypsin_: n_rhOPN_ ratio of 5:1 is recommended for the digestion and identification of 100 ng/mL rhOPN. If comparing the results of 4 μg/mL and 100 ng/mL digested rhOPN it could also be seen that in order to obtain more peptides, the ratio of n_trypsin_: n_rhOPN_ should be increased when rhOPN concentration is decreased. Thus, it is important to optimize the molar ratios of trypsin to protein for the digestion to get satisfying results for further analysis. A lower ratio of n_trypsin_: n_protein_ is preferred if only the main peak is interesting, while higher ratio is needed if the identification should be carried out by Mascot data search using all MS ions.

### Protein A and DEAE-CB effects on protein reference samples

**Fig 4:**
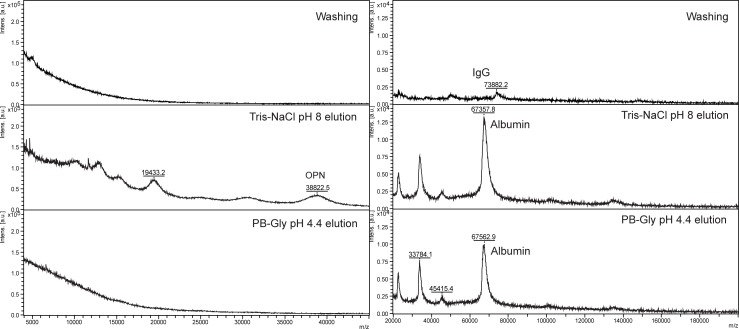
MALDI-MS of proteins in different extraction fractions using DEAE-CB. Left: rhOPN (200 μg/mL). Right: protein mixture (BSA 40 mg/mL, IgG 10 mg/mL, rhOPN 200 μg /mL).

Protein A and DEAE-CB were evaluated separately for rhOPN reference samples or protein mixtures in order to investigate and optimize the depletion of IgG and albumin. It was found that BSA and rhOPN were partially bound to protein A, while IgG could be bound completely (S4 Fig). On the other hand, BSA and rhOPN were strongly bound to DEAE-CB, but IgG was not bound. Thus, the depletion of IgG could be achieved by using DEAE-CB in the first step. BSA could be eluted both by Tris-NaCl pH 8.0 and PB-Gly pH 4.4, while rhOPN only appeared in the Tris-NaCl pH 8.0 fraction (Fig 4). In Fig 4 (right), the detection of rhOPN is obstructed by the high intensity albumin peaks and could not be seen clearly. Thus, the depletion of albumin is important. The separation of albumin and rhOPN was further studied with rhOPN in human plasma samples by the following elution order: PB-Gly 4.4 buffer for albumin elution (twice), followed by Tris-NaCl 8.0 to elute rhOPN.

### rhOPN extraction from human plasma using DEAE-CB

**Fig 5:**
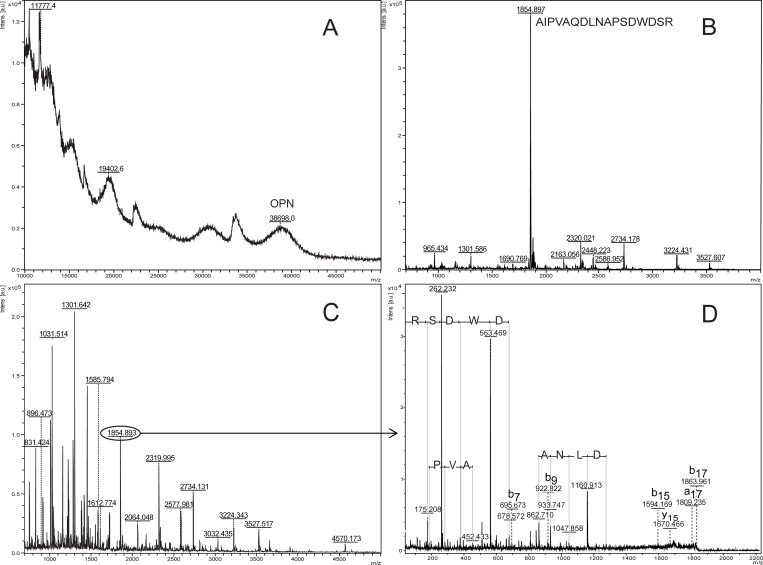
MALDI-MS and MS/MS of Elution fraction 3, from human plasma samples. (A) Spiked with 1 mg/mL rhOPN, intact protein. (B) Spiked with 1 mg/mL rhOPN, trypsin digest. (C) Spiked with 100 μg/mL rhOPN, trypsin digest. (D) Spiked with 100 μg/mL rhOPN, MS/MS on *m/z* 1854.898 from trypsin digest.

MALDI-MS analysis of human plasma samples spiked with 1 mg/mL rhOPN showed that rhOPN was eluted in Elution fraction 3 (Fig 5A), and could be separated from most of the plasma proteins. MS spectra of Elution fraction 3 digests were compared to digests from plasma without rhOPN spiking (S5 Fig), and rhOPN reference sample digests. Peptides originating from rhOPN digests were the only peptides that could be seen in Elution fraction 3, with *m/z* 1854.898 being the main peak (Fig 5B). The digests could also be identified as human OPN using Biotools (S6 Fig). However, when decreasing the rhOPN concentration to 100 μg/mL in the plasma, some peaks originating from other digested proteins began to appear in the MS spectra, and the identification of OPN was obstructed (Fig 5C). Thus, the MS/MS fragmentation of peak *m/z* 1854.898 was analyzed resulting in successful identification as human OPN, with *m/z* 262.151, 563.257, 1160.595 as major fragments. Identification results obtained from Biotools are shown in Fig 5D and S7 Fig. There is no glycosylation site in the fingerprint peptide (AIPVAQDLNAP**S**DWD**S**R) with *m/z* 1854.898, but two possible phosphorylation sites (bold S) [9, 45–47]. However, the dephosphorylation studies on 100 μg/mL rhOPN both before and after trypsin digestion show no phosphorylation in this peptide (S8 Fig). First of all, no phosphorylated peptide with mass 1934.878 or 2014.838 (1854.898 + 79.980 or 1854.898 + 159.960) can be detected. Second, there was no change in peak intensity for the fingerprint peptide with and without dephosphorylation. Therefore, in the following studies, the analysis of rhOPN was focused on the fingerprint peptide with *m/z* 1854.898. In order to detect and identify lower concentrations of rhOPN in plasma, in an attempt to reach the levels found in healthy people´s or patients’ plasma, optimization of the buffer systems and the MALDI matrices was carried out.

#### Binding and elution buffer evaluation

**Fig 6:**
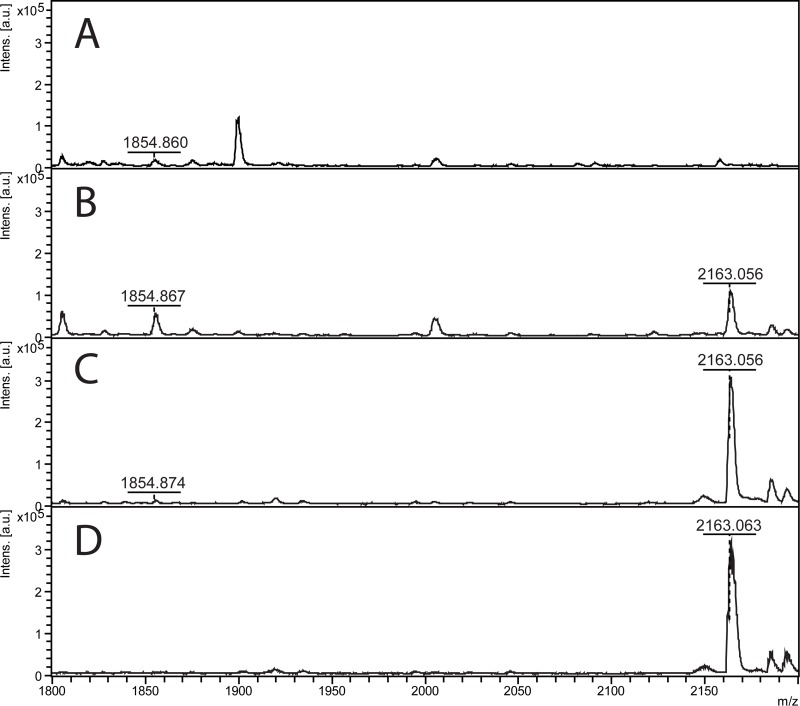
MALDI-MS of trypsin digests of elution fractions with 20 μg/ml rhOPN in human plasma. (A) PB-Gly pH 4.4 + 0.1 M NaCl, (B) PB-Gly pH 4.4 + 0.2 M NaCl, (C) PB-Gly pH 4.4 + 0.3 M NaCl, (D) PB-Gly pH 4.4 + 0.4 M NaCl.

It has been reported that a lower pH is better for protein binding on CB, and that separation could be achieved by increasing ionic strength in the elution buffers [48, 49]. In order to accomplish a better separation from plasma proteins, it was necessary to establish the salt concentration at which rhOPN could be eluted. Comparisons were therefore carried out using 10 mM Tris-HCl pH 8.0 and PB-Gly pH 4.4 buffers with increased NaCl concentrations for elution of 20 μg/ml rhOPN in human plasma samples. When the six consecutive digested elution fractions were analyzed, rhOPN was mainly recovered in the fraction containing PB-Gly pH 4.4 + 0.2 M NaCl, and some in the fraction containing 0.3 M NaCl (Fig 6). However, when using Tris-HCl buffers the rhOPN fingerprint peptide was spread out in totally four elution fractions with NaCl concentration ranging from 0.1 to 0.4 M (S9 Fig). Moreover, very bad S/N values (<3) were obtained when using only 10 mM Tris-HCl, pH 8.0 + 0.4 M NaCl in Elution fraction 3 to elute a plasma sample with 4 μg/ml rhOPN. So, in following studies, Tris-NaCl pH 8.0 or 0.2 M NaH_2_PO_4_ + 0.2 M Glycine + 0.3 M NaCl, pH 4.4 (PB-Gly-NaCl pH 4.4) was used in Elution fraction 3 for further comparison.

First of all, studies on 20 μg/ml rhOPN in human plasma samples were carried out using 10 mM Tris-HCl and 10 mM NaH_2_PO_4_, pH 4.0 and 8.0 respectively as the binding buffer, the elution order was twice with PB-Gly pH 4.4, followed by one elution with Tris-NaCl pH 8.0. Results represented by S/N values showed that higher S/N values could be obtained for peak *m/z* 1854.898 when using a buffer with the lower pH (Table 2 and S10 Fig). Furthermore, it was observed that the S/N ratio of peak *m/z* 1854.898 in relation to an interfering peak at around *m/z* 1847 also was increased when using NaH_2_PO_4_ buffers. Therefore, NaH_2_PO_4_ buffers were selected for further optimization of buffer pH and concentration, regarding binding efficiency, instead of Tris-HCl buffers.

**Table 2:** MALDI-MS S/N data on peak *m/z* 1854.898 of trypsin digests of Elution fraction 3, from plasma samples, using different binding buffers.

	Peak 1854.898 S/N	S/N ratio 1854.898/1847
10 mM NaH_2_PO_4_ pH 4.0	25.2 ± 6.2	3.1 ± 0.8
10 mM NaH_2_PO_4_ pH 8.0	17.5 ± 5.0	2.0 ± 0.3
10 mM Tris-HCl pH 4.0	17.5 ± 3.6	2.3 ± 0.4
10 mM Tris-HCl pH 8.0	13.0 ± 5.2	1.8 ± 0.4

All samples are human plasma samples spiked with 20 μg/ml rhOPN. MS peak *m/z* 1854.898 originates from rhOPN and *m/z* 1847 from an interfering protein. n = 6, 2 samples, 3 spots for each. MALDI matrix: DHB

NaH_2_PO_4_ binding buffers with different pH (4.0, 6.0 and 8.0) and concentration (10 mM and 100 mM) were investigated for 2 μg/ml and 4 μg/ml rhOPN in human plasma. The choice of these three pH values was based on the isoelectric points (pI) of OPN and the abundant proteins: 3.5-4.1 for OPN [50] (theoretical 4.4 for rhOPN [51]), around 5.4–5.8 for HSA [52] and 6.6–10.0 for IgG [52]. The idea was to see if the interaction between proteins and DEAE-CB would be affected by the pH. Tris-NaCl pH 8.0 and PB-Gly-NaCl pH 4.4 were also evaluated as buffers for the third elution fraction. The results as MALDI-TOF MS S/N values of peak *m/z* 1854.898 are shown in Table 3. The data suggest that a more effective elution of rhOPN could be obtained when using PB-Gly-NaCl pH 4.4 compared to Tris-NaCl pH 8.0, no matter the concentration or pH of the binding buffer NaH_2_PO_4_. The highest average S/N of the peak at *m/z* 1854.898 for 4 μg/mL rhOPN in plasma was 24.4, obtained by binding with 100 mM NaH_2_PO_4_ pH 4.0 and eluting with PB-Gly-NaCl pH 4.4. For elution with Tris-NaCl 8.0, the highest S/N was 11.3, obtained by binding with 100 mM NaH_2_PO_4_ pH 6.0. Binding of rhOPN (2 or 4 μg/mL in plasma samples) was improved by an increase in buffer concentration. No significant difference was observed when comparing binding buffers with different pH values for plasma samples with 4 μg/ml rhOPN added, but the improvement of rhOPN binding at pH 4.0 was obvious for 2 μg/ml rhOPN. The highest average S/N value (10.2) for the peak at *m/z* 1854.898 at a concentration of 2 μg/ml rhOPN was obtained when binding with 100mM NaH_2_PO_4_ pH 4.0, and elution with PB-Gly-NaCl pH 4.4 for the third elution fraction was used. Therefore, for the studies of 1 μg/ml rhOPN in plasma samples, 100 mM NaH_2_PO_4_ pH 4.0 buffer was used for binding and washing.

**Table 3:** MALDI-MS S/N data on peak *m/z* 1854.898 of trypsin digests of Elution fraction 3, from plasma samples, using different binding and elution buffers.

Binding buffer		Elution Tris-NaCl 8	Elution PB-Gly-NaCl 4.4	Elution PB-Gly-NaCl 4.4
		4 μg/mL rhOPN in plasma	4 μg/mL rhOPN in plasma	2 μg/mL rhOPN in plasma
10 mM NaH_2_PO_4_	pH 4.0	NA	13.9 ± 4.2 (n = 8)	3.8 ± 1.8 (n = 5)
	pH 6.0	4.0 ± 2.0 (n = 4)	16.6 ± 3.4 (n = 8)	NA
	pH 8.0	6.0 ± 3.6 (n = 4)	12.0 ± 5.5 (n = 8)	ND (n = 5)
100 mM NaH_2_PO_4_	pH 4.0	6.3 ± 2.9 (n = 4)	24.4 ± 5.4 (n = 5)	10.2 ± 3.9 (n = 15)
			10.6 ± 2.7 (n = 10) denatured	4.9 ± 1.4 (n = 10) denatured
	pH 6.0	11.3 ± 4.3 (n = 4)	18.6 ± 7.1 (n = 5)	5.6 ± 3.7 (n = 5)
	pH 8.0	5.8 ± 2.2 (n = 4)	21.4 ± 1.5 (n = 5)	4.6 ± 2.7 (n = 10)

NA–Not analyzed. ND–Not detected. For the data having S/N <3, 3 was used for calculation (Raw data are shown in S2 Table). All samples were without denaturation except the ones marked with denatured. n = 4 ~ 15, from 1 ~ 3 samples. MALDI matrix: DHB

S/N data of the peak at *m/z* 1854.898 were also compared with denatured samples in Table 3. However, for both 2 and 4 μg/mL rhOPN in plasma samples, S/N decreased a lot when the Elution fraction 3 was denatured before digestion. The use of high temperatures for denaturation brings a risk for protein precipitation, which would cause a decrease in the S/N. Therefore, no denaturation was carried out in further studies.

The pH of the elution buffer 0.2 M NaH_2_PO_4_ + 0.2 M Glycine + 0.3 M NaCl (PB-Gly-NaCl) was also investigated. For the recovery of rhOPN (1 μg/ml) in human plasma PB-Gly-NaCl pH 4.4 and pH 8.0 were compared. The peak at *m/z* 1854.9 obtained when using PB-Gly-NaCl pH 8.0 had an average S/N value less than 3, while it was 5.4 (n = 8, 2 samples, 4 spots each) recovered by PB-Gly-NaCl pH 4.4 (S11 Fig).

In summary, 100 mM NaH_2_PO_4_ pH 4.0 for binding and 0.2 M NaH_2_PO_4_ + 0.2 M Glycine + 0.3 M NaCl pH 4.4 for elution was concluded to be the most suitable buffers for recovery of low concentrations of rhOPN in plasma samples.

#### Trypsin digestion of rhOPN in human plasma samples

In a previous section the amount of trypsin was shown to have important effects on rhOPN digestion for the same amount of rhOPN samples. The MALDI-TOF MS spectra of Elution fraction 3 from 1–2 μg/ml rhOPN in human plasma digested using 0.25 and 0.5 μg trypsin are shown in S12 Fig. No intact protein could be seen in the spectra when using 0.5 μg trypsin for digestion. However, 0.25 μg trypsin was not enough for the complete digestion of Elution fraction 3 from 1 μg/ml rhOPN in human plasma samples since the plasma amount was doubled. This can be illustrated by the appearance of a small albumin peak in the spectrum. When using different amounts of trypsin for digests of 2 μg/mL rhOPN in plasma the S/N data on peak *m/z* 1854.898 show no considerable difference (Table 4). However, for 1 μg/ml rhOPN in plasma samples the S/N values obtained when using 0.5 μg trypsin were increased compared to 1 μg trypsin. A possible reason for this is that a decreased interference from peaks originating from digested albumin would affect the detection of the rhOPN fingerprint peptide. Nonetheless, S/N values decreased for 1 μg/mL rhOPN in plasma samples when using 0.25 μg trypsin for digestion compared to 0.5 μg trypsin. This could be due to incomplete digestion of rhOPN. According to the results shown in Table 4, 0.5 μg trypsin could be the best choice for digestion.

**Table 4:** MALDI-MS S/N data on peak *m/z* 1854.898 of trypsin digests of Elution fraction 3, from plasma samples, using different amounts of trypsin.

Trypsin amount	2 μg/mL rhOPN in plasma	1 μg/mL rhOPN in plasma
1 μg	10.2 ± 3.9 (n = 15)	5.4 ± 2.5 (n = 8)
0.5 μg	9.5 ± 1.5 (n = 6)	8.2 ± 2.3 (n = 6)
0.25 μg	11.8 ± 5.2 (n = 6)	7.0 ± 1.8 (n = 6)

For the data having S/N <3, 3 was used for calculation (Raw data are shown in S3 Table). All samples were bound with 100mM NaH_2_PO_4_ pH 4.0, and eluted with PB-Gly-NaCl pH 4.4. n = 6 ~ 15, from 1 ~ 3 samples. MALDI matrix: DHB

#### MALDI matrix evaluation

**Fig 7:**
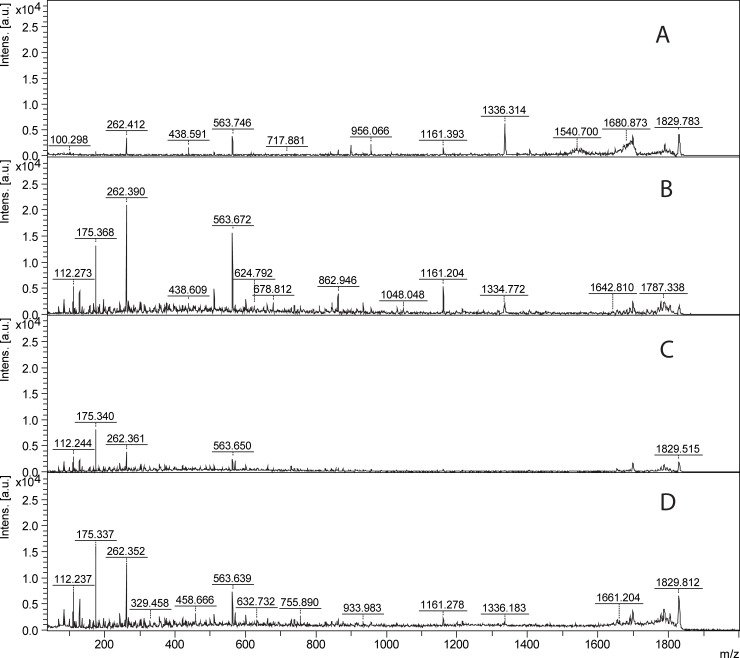
MALDI-TOF MS/MS on the peak at *m/z* 1854.898, trypsin digests of Elution fraction 3. (A) 2 μg/ml rhOPN in human plasma, DHB, (B) 2 μg/ml rhOPN in human plasma, saturated HCCA, (C) 1 μg/ml rhOPN in human plasma, 2.8 mg/mL HCCA, and (D) 1 μg/ml rhOPN in human plasma, saturated HCCA.

An important factor when analyzing peptides with MALDI-MS is the choice of matrix. Therefore, the commonly used matrices DHB and HCCA were investigated both in MS and MS/MS modes for 2 μg/ml and 4 μg/ml rhOPN in plasma samples using 100 mM NaH_2_PO_4_ and PB-Gly-NaCl pH 4.4 as binding and elution buffers respectively. Both the peak intensity and S/N values for the peak at *m/z* 1854.898 were considerably decreased when using HCCA saturated in TA 30 instead of 20 mg/ml DHB in TA 30 in MS mode (Table 5). However, the performance of HCCA was better when compared to DHB for the fragments obtained from the peptide *m/z* 1854.898 in MS/MS mode for 2 μg/ml rhOPN in plasma samples (Fig 7A and 7B). The selected parent ion was well fragmented using HCCA and a number of fragments with low *m/z* could be seen. Fewer fragments and broad peaks were observed in the higher *m/z* range of the MS/MS spectra, though. The performance differences of the matrices in MS vs MS/MS mode is likely due to the higher plume density of HCCA yielding more fragmentation as ions are accelerated in the MS/MS mode [53]. In addition to saturated HCCA, a concentration of 2.8 mg/ml HCCA was also evaluated in MS/MS mode for 1 μg/ml rhOPN in plasma samples (Fig 7C and 7D). The peak intensities of the main fragments from the parent ion 1854.898 were higher when saturated HCCA was used compared to 2.8 mg/ml HCCA. Better identification results were also obtained when using saturated HCCA compared to DHB or 2.8 mg/mL HCCA (S13 Fig).

**Table 5:** MALDI-MS S/N data on peak *m/z* 1854.898 of trypsin digests of Elution fraction 3, from plasma samples, using different MALDI matrices.

Matrix	Binding buffer		4 μg/mL rhOPN in plasma	2 μg/mL rhOPN in plasma
DHB 20mg/mL	100 mM NaH_2_PO_4_	pH 4.0	24.4 ± 5.4 (n = 5)	10.2 ± 3.9 (n = 15)
		pH 6.0	18.6 ± 7.1 (n = 5)	5.6 ± 3.7 (n = 5)
		pH 8.0	21.4 ± 1.5 (n = 5)	4.6 ± 2.7 (n = 10)
HCCA saturated	100 mM NaH_2_PO_4_	pH 4.0	3.6 ± 1.3 (n = 5)	5.0 ± 1.4 (n = 5)
		pH 6.0	4.2 ± 1.8 (n = 5)	NA
		pH 8.0	5.6 ± 3.3 (n = 5)	ND (n = 5)

NA–Not analyzed. ND–Not detected. For the data having S/N <3, 3 was used for calculation (Raw data are shown in S4 Table).

Plasma samples without addition of rhOPN were treated with DEAE-CB using 100 mM NaH_2_PO_4_ pH 4.0 for binding and washing, and PB-Gly-NaCl pH 4.4 as the third elution fraction buffer. No OPN fingerprint peak at *m/z* 1854.898 could be detected in the MS mode using DHB or in the MS/MS mode using saturated HCCA as matrix. The plasma concentration of human OPN in healthy persons is in the range from <7.8 to 87 ng/mL [16]. Here, a concentration of 1 μg/ml could be detected. Hence, to make detection of the lowest concentration of human OPN in healthy people possible, the present method needs to be supplemented by an extra concentration step *e*.*g*. using chip based isoelectric focusing [54]. This is outside the scope of the present study, though. Nevertheless, this DEAE-CB antibody-free isolation method could still potentially be used for human OPN as a biomarker for diseases where the concentration is raised to higher than 1 μg/ml, such as some cancers and heart diseases mentioned in the introduction section. This method could also be used instead of immunoassays for the preconcentration of OPN in human plasma.

## Conclusions

Selective binding to and elution from DEAE-CB were used successfully for the depletion of IgG and albumin in human plasma in order to facilitate the detection of rhOPN. The separation of OPN and albumin could be achieved by optimizing the elution buffer system. Albumin could be eluted using the buffer PB-Gly pH 4.4, while rhOPN was recovered using PB-Gly pH 4.4 containg 0.3 M NaCl. The analysis of an OPN fingerprint peptide with *m/z* 1854.898 was utilized for detection in MS mode using DHB as the matrix, and its fragments were detected in MS/MS mode using saturated HCCA. By this method, 1 μg/ml rhOPN could be isolated from abundant proteins in human plasma and identified as human OPN. A quite low volume of plasma, only 5 μL to 10 μL was needed for the whole procedure using the described method.

## Supporting information

S1 Table Concentrations and volumes of rhOPN, trypsin and salt in rhOPN reference samples for tryptic digestion. (PDF)

S2 Table MALDI-MS S/N data on peak *m/z* 1854.898 of trypsin digests of Elution fraction 3, from plasma samples, using different binding and elution buffers. 4 μg/mL and 2 μg/mL rhOPN in human plasma samples were investigated, using matrix DHB. NA–Not analyzed. ND–Not detected.
(PDF)

S3 Table MALDI-MS S/N data on peak m/z 1854.898 of trypsin digests of Elution fraction 3, from plasma samples, using different amount of trypsin. 1 μg/mL and 2 μg/mL rhOPN in human plasma samples were investigated, using matrix DHB. All samples were bound with 100mM NaH2PO4 pH 4.0, and eluted with PB-Gly-NaCl pH 4.4.
(PDF)

S4 Table MALDI-MS S/N data on peak *m/z* 1854.898 of trypsin digests of Elution fraction 3, from plasma samples, using different matrices. 4 μg/mL and 2 μg/mL rhOPN in human plasma samples were investigated using different binding buffers and MALDI matrices. NA–not analyzed. ND–Not detected.
(PDF)

S1 Fig Biotools Mascot identification results for 100 ng/mL digested rhOPN reference sample. (A) Score for identification. (B) Identified human OPN digests and their corresponding sequences.
(PDF)

S2 Fig Biotools Mascot identification results for MS ions of trypsin digest from 4 μg/ml rhOPN reference samples. (A) n_trypsin_: n_rhOPN_ = 1:25. (B) n_trypsin_: n_rhOPN_ = 1:5
(PDF)

S3 Fig MALDI-TOF MS of 200 ng/mL rhOPN trypsin digests with different molar ratio of trypsin to rhOPN. (A) n_trypsin_: n_rhOPN_ = 1:1. (B) n_trypsin_: n_rhOPN_ = 5:1
(PDF)

S4 Fig MALDI-TOF MS of proteins in different extraction fractions using Protein A as affinity material. Left: rhOPN (200 μg/mL). Right: protein mixture (BSA 40 mg/mL, IgG 10 mg/mL, rhOPN 200 μg/mL).
(PDF)

S5 Fig MALDI-MS of trypsin digests of plasma samples. (A) trypsin digests of Elution fraction 3 from 1 mg/mL rhOPN in human plasma sample. (B) trypsin digests of pure human plasma. (C) Comparison of the main peak *m/z* 1854.898 by superimposing spectrum (A) and (B).
(PDF)

S6 Fig Biotools Mascot identification results for MS ions of trypsin digest of Elution fraction 3, extracted from rhOPN (100 μg/mL) in human plasma. (A) Score for identification. (B) Identified peptides and their corresponding sequences in human OPN.
(PDF)

S7 Fig Biotools Mascot identification results for MS/MS on the peak at m/z 1854.898 of trypsin digest from Elution fraction 3, extracted from rhOPN (100 μg/mL) in human plasma. (A) Score for identification. (B) Identified MS/MS fragments of peak m/z 1854.898 and their corresponding sequences in human OPN.
(PDF)

S8 Fig **MALDI-TOF MS of 100** μ**g/mL rhOPN trypsin digests:** (A) without dephosphorylation. (B) and (C) dephosphorylation before digestion. (D) and (E) dephosphorylation after digestion. (B) and (D) 0.25 unit phosphatase. (C) and (E) 1 unit phosphatase.
(PDF)

S9 Fig MALDI-MS of trypsin digests from elution fractions with 20 μg/ml rhOPN in human plasma. (A) 10 mM Tris-HCl pH 8 + 0.1 M NaCl, (B) 10 mM Tris-HCl pH 8 + 0.2 M NaCl, (C) 10 mM Tris-HCl pH 8 + 0.3 M NaCl, (D) 10 mM Tris-HCl pH 8 + 0.4 M NaCl, (E) 10 mM Tris-HCl pH 8 + 0.5 M NaCl.
(PDF)

S10 Fig MALDI-MS spectra for digested rhOPN, 20 μg/ml added in a human plasma sample, elution fraction 3. (A) Binding buffer 10 mM NaH_2_PO_4_ pH 4. (B) Binding buffer 10 mM NaH_2_PO_4_ pH 8. (C) Binding buffer 10 mM Tris-HCl pH 4. (D) Binding buffer 10 mM Tris-HCl pH 8.
(PDF)

S11 Fig MALDI-MS spectra for digested rhOPN, 1 μg/ml added in a human plasma sample, elution fraction 3. (A) Eluted by PB-Gly-NaCl pH 4.4. (B) Eluted by PB-Gly-NaCl pH 8.
(PDF)

S12 Fig MALDI-TOF MS of Elution fraction 3 from 1–2 μg/ml rhOPN in human plasma. (A) 2 μg/ml rhOPN, 0.5 μg trypsin. (B) 1 μg/ml rhOPN, 0.5 μg trypsin. (C) 2 μg/ml rhOPN, 0.25 μg trypsin. (D) 1 μg/ml rhOPN, 0.25 μg trypsin.
(PDF)

S13 Fig Biotools Mascot identification results for MS/MS on the peak at m/z 1854.898 of trypsin digest from Elution fraction 3, extracted from plasma samples. (A) rhOPN (2 μg/ml) in human plasma, DHB. (B) rhOPN (2 μg/ml) in human plasma, saturated HCCA. (C) rhOPN (1 μg/ml) in human plasma, 2.8 mg/mL HCCA. (D) rhOPN (1 μg/ml) in human plasma, saturated HCCA. (E) Identified MS/MS fragments of peak m/z 1854.898 from (D) and their corresponding sequences in human OPN.
(PDF)
