# Supplementary material for: An antibody-free sample pretreatment method for osteopontin combined with MALDI-TOF MS/MS analysis
Source: PLoS One. 2019 Mar 7;14(3):e0213405. doi: 10.1371/journal.pone.0213405 (PMC6405093; doi:10.1371/journal.pone.0213405)
Supplement: S1 Table — (PDF) [file pone.0213405.s001.pdf]

S1 Table. Concentrations and volumes of rhOPN, trypsin and salt in rhOPN reference samples for tryptic digestion.

$n_{trypsin}$: n_{rhOPN}	**Trypsin** Conc.	volume	**rhOPN** Conc.	volume	**NH_4HCO_3** Conc.	volume	**MQ H_2O** volume	**Total volume**
1:25	26.3 nM	1.5 µL	4 µg/mL (103 nM)	10 µL	40 mM	7.5 µL	11 µL	30 µL
1:5	26.3 nM	7.5 µL	4 µg/mL (103 nM)	10 µL	40 mM	7.5 µL	5.0 µL	30 µL
1:4	26.3 nM	7.5 µL	2 µg/mL (52 nM)	15 µL	40 mM	7.5 µL	0.0 µL	30 µL
1:1	26.3 nM	6.0 µL	400 ng/mL (10 nM)	15 µL	40 mM	7.5 µL	1.5 µL	30 µL
1:1	26.3 nM	9.5 µL	200 ng/mL (5.2 nM)	50 µL	40 mM	25 µL	15.5 µL	100 µL
5:1	263 nM	4.8 µL	200 ng/mL (5.2 nM)	50 µL	40 mM	25 µL	20.2 µL	100 µL
5:1	420 nM	1.5 µL	100 ng/mL (2.6 nM)	50 µL	40 mM	25 µL	23.5 µL	100 µL
10:1	420 nM	3.0 µL	100 ng/mL (2.6 nM)	50 µL	40 mM	25 µL	22.0 µL	100 µL
