# Supplementary material for: An antibody-free sample pretreatment method for osteopontin combined with MALDI-TOF MS/MS analysis
Source: PLoS One. 2019 Mar 7;14(3):e0213405. doi: 10.1371/journal.pone.0213405 (PMC6405093; doi:10.1371/journal.pone.0213405)
Supplement: S2 Table — 4 μg/mL and 2 μg/mL rhOPN in human plasma samples were investigated, using matrix DHB. NA–Not analyzed. ND–Not detected. (PDF) [file pone.0213405.s002.pdf]

S2 Table. MALDI-MS S/N data on peak *m/z* 1854.898 of trypsin digests of Elution fraction 3, from plasma samples, using different binding and elution buffers.

Binding buffer		Eltuion Tris-NaCl pH 8<br><br>**4 µg/mL** rhOPN in plasma	Elution PB-Gly-NaCl, pH 4.4<br><br>**4 µg/mL** rhOPN in plasma	Elution PB-Gly-NaCl, pH 4.4<br><br>**2 µg/mL** rhOPN in plasma
10mM NaH_2PO_4	pH 4	NA	16, 9, 20, 11, 11, 19, 10, 15	7, 3, <3, <3, 3
	pH 6	3, 7, 3, <3	18, 22, 21, 14, 16, 14, 13, 15	NA
	pH 8	6, 4, 11, 3	8, 8, 17, 11, 13, 24, 14, 11	ND
100mM NaH_2PO_4	pH 4	3, 10, 6, 6	29, 15, 26, 26, 26	8, 19, 9, 10, 9, 7, 6, 7, 6, 11, 6, 13, 13, 16, 13
			11, 11, 10, 9, 7, 17, 8, 11, 11, 11 (denatured)	4, 5, 3, 4, 3, 6, 5, 6, 7, 6 (denatured)
	pH 6	10, 6, 13, 16	17, 8, 24, 18, 26	8, 11, 3, <3, 3
	pH 8	<3, 5, 8, 7	24, 21, 21, 21, 20	3, <3, <3, 10, 9, <3, 4, 5, <3, <3

4 µg/mL and 2 µg/mL rhOPN in human plasma samples were investigated, using matrix DHB. NA – Not analyzed. ND – Not detected. All samples were without denaturation except the ones marked with denatured.
