# Supplementary material for: An antibody-free sample pretreatment method for osteopontin combined with MALDI-TOF MS/MS analysis
Source: PLoS One. 2019 Mar 7;14(3):e0213405. doi: 10.1371/journal.pone.0213405 (PMC6405093; doi:10.1371/journal.pone.0213405)
Supplement: S3 Table — 1 μg/mL and 2 μg/mL rhOPN in human plasma samples were investigated, using matrix DHB. All samples were bound with 100mM NaH2PO4 pH 4.0, and eluted with PB-Gly-NaCl pH 4.4. (PDF) [file pone.0213405.s003.pdf]

S3 Table. MALDI-MS S/N data on peak *m/z* 1854.898 of trypsin digests of Elution fraction 3, from plasma samples, using different amount of trypsin.

Trypsin amount	2 µg/mL rhOPN in plasma	1 µg/mL rhOPN in plasma
1 µg	8, 19, 9, 10, 9, 7, 6, 7, 6, 11, 6, 13, 13, 16, 13	7, <3, 3, 10, 7, 5, 5, <3
0.5 µg	10, 8, 12, 9, 10, 8	9, 7, 6, 12, 6, 9
0.25 µg	8, 19, 7, 7, 14, 16	7, 4, 6, 8, 8, 9

1 µg/mL and 2 µg/mL rhOPN in human plasma samples were investigated, using matrix DHB. All samples were bound with 100mM NaH_2PO_4 pH 4.0, and eluted with PB-Gly-NaCl pH 4.4.
