# Supplementary material for: An antibody-free sample pretreatment method for osteopontin combined with MALDI-TOF MS/MS analysis
Source: PLoS One. 2019 Mar 7;14(3):e0213405. doi: 10.1371/journal.pone.0213405 (PMC6405093; doi:10.1371/journal.pone.0213405)
Supplement: S4 Table — 4 μg/mL and 2 μg/mL rhOPN in human plasma samples were investigated using different binding buffers and MALDI matrices. NA–not analyzed. ND–Not detected. (PDF) [file pone.0213405.s004.pdf]

S4 Table. MALDI-MS S/N data on peak *m/z* 1854.898 of trypsin digests of Elution fraction 3, from plasma samples, using different matrices.

Matrix	Binding buffer		4 µg/mL rhOPN in plasma	2 µg/mL rhOPN in plasma
DHB 20mg/mL	100 mM NaH_2PO_4	pH 4.0	29, 15, 26, 26, 26	8, 19, 9, 10, 9, 7, 6, 7, 6, 11, 6, 13, 13, 16, 13
		pH 6.0	17, 8, 24, 18, 26	8, 11, 3, <3, 3
		pH 8.0	24, 21, 21, 21, 20	3, <3, <3, 10, 9, <3, 4, 5, <3, <3
HCCA Saturated	100 mM NaH_2PO_4	pH 4.0	<3, 6, <3, <3, <3	5, <3, 6, 6, <3
		pH 6.0	<3, 7, 5, <3, <3	NA
		pH 8.0	6, 11, 5, <3, <3	ND

4 µg/mL and 2 µg/mL rhOPN in human plasma samples were investigated using different binding buffers and MALDI matrices. NA – not analyzed. ND – Not detected.
