# Supplementary material for: An antibody-free sample pretreatment method for osteopontin combined with MALDI-TOF MS/MS analysis
Source: PLoS One. 2019 Mar 7;14(3):e0213405. doi: 10.1371/journal.pone.0213405 (PMC6405093; doi:10.1371/journal.pone.0213405)
Supplement: S1 Fig — (A) Score for identification. (B) Identified human OPN digests and their corresponding sequences. (PDF) [file pone.0213405.s005.pdf]

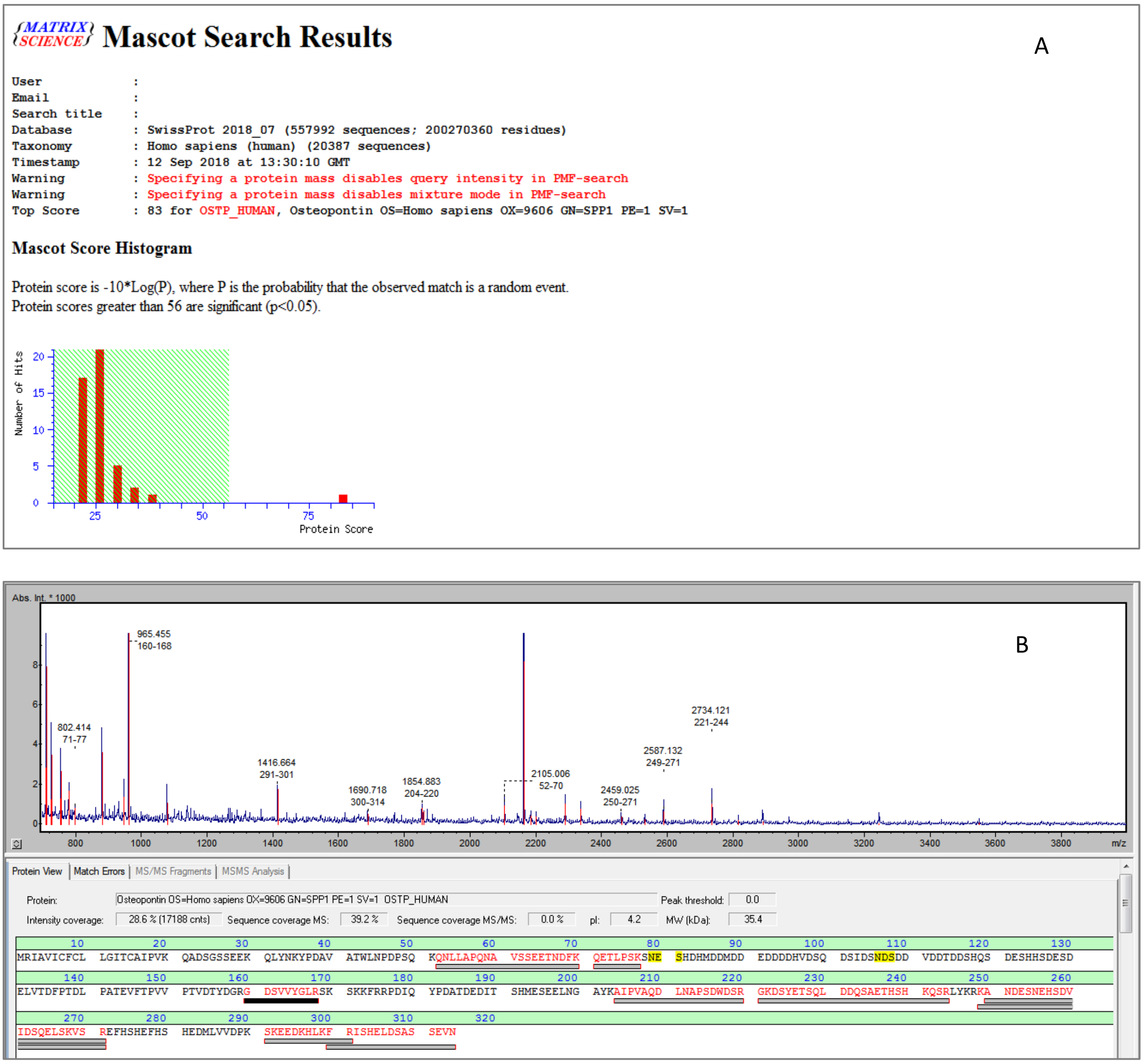

S1 Fig. Biotools Mascot identification results for 100 ng/mL digested rhOPN reference sample. (A) Score for identification. (B) Identified human OPN digests and their corresponding sequences.
