# Supplementary material for: An antibody-free sample pretreatment method for osteopontin combined with MALDI-TOF MS/MS analysis
Source: PLoS One. 2019 Mar 7;14(3):e0213405. doi: 10.1371/journal.pone.0213405 (PMC6405093; doi:10.1371/journal.pone.0213405)
Supplement: S2 Fig — (A) ntrypsin: nrhOPN = 1:25. (B) ntrypsin: nrhOPN = 1:5 (PDF) [file pone.0213405.s006.pdf]

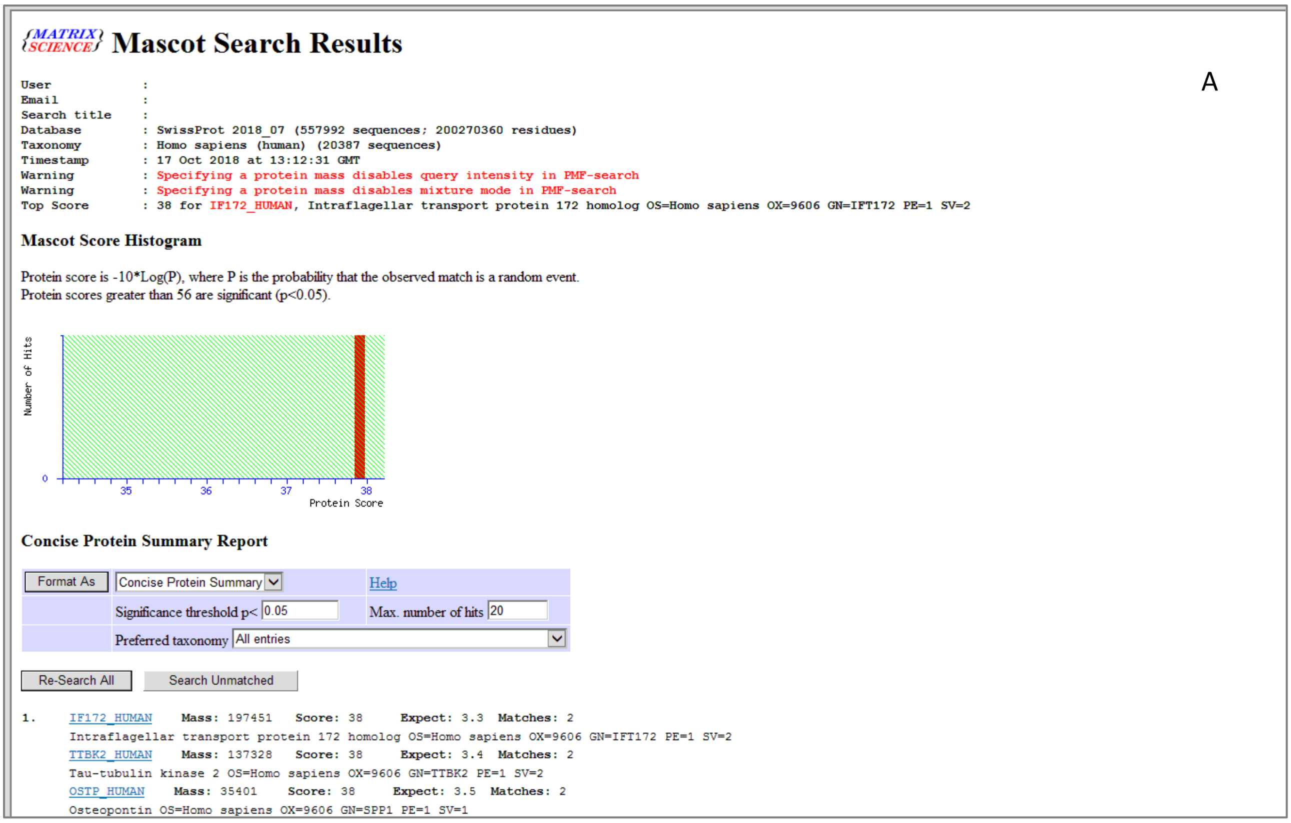

A

Mascot Search Results

```
User          :
Email         :
Search title  :
Database      : SwissProt 2018_07 (557992 sequences; 200270360 residues)
Taxonomy      : Homo sapiens (human) (20387 sequences)
Timestamp     : 17 Oct 2018 at 13:12:31 GMT
Warning       : Specifying a protein mass disables query intensity in PMF-search
Warning       : Specifying a protein mass disables mixture mode in PMF-search
Top Score     : 38 for IF172_HUMAN, Intraflagellar transport protein 172 homolog OS=Homo sapiens OX=9606 GN=IFT172 PE=1 SV=2
```

Mascot Score Histogram

Protein score is -10*Log(P), where P is the probability that the observed match is a random event.
Protein scores greater than 56 are significant (p<0.05).

Concise Protein Summary Report

Format As | Concise Protein Summary | Help
Significance threshold p< 0.05 | Max. number of hits 20
Preferred taxonomy All entries

Re-Search All | Search Unmatched

```
1.  IF172_HUMAN   Mass: 197451   Score: 38   Expect: 3.3   Matches: 2
    Intraflagellar transport protein 172 homolog OS=Homo sapiens OX=9606 GN=IFT172 PE=1 SV=2
    TTBK2_HUMAN   Mass: 137328   Score: 38   Expect: 3.4   Matches: 2
    Tau-tubulin kinase 2 OS=Homo sapiens OX=9606 GN=TTBK2 PE=1 SV=2
    OSTP_HUMAN   Mass: 35401   Score: 38   Expect: 3.5   Matches: 2
    Osteopontin OS=Homo sapiens OX=9606 GN=SPP1 PE=1 SV=1
```

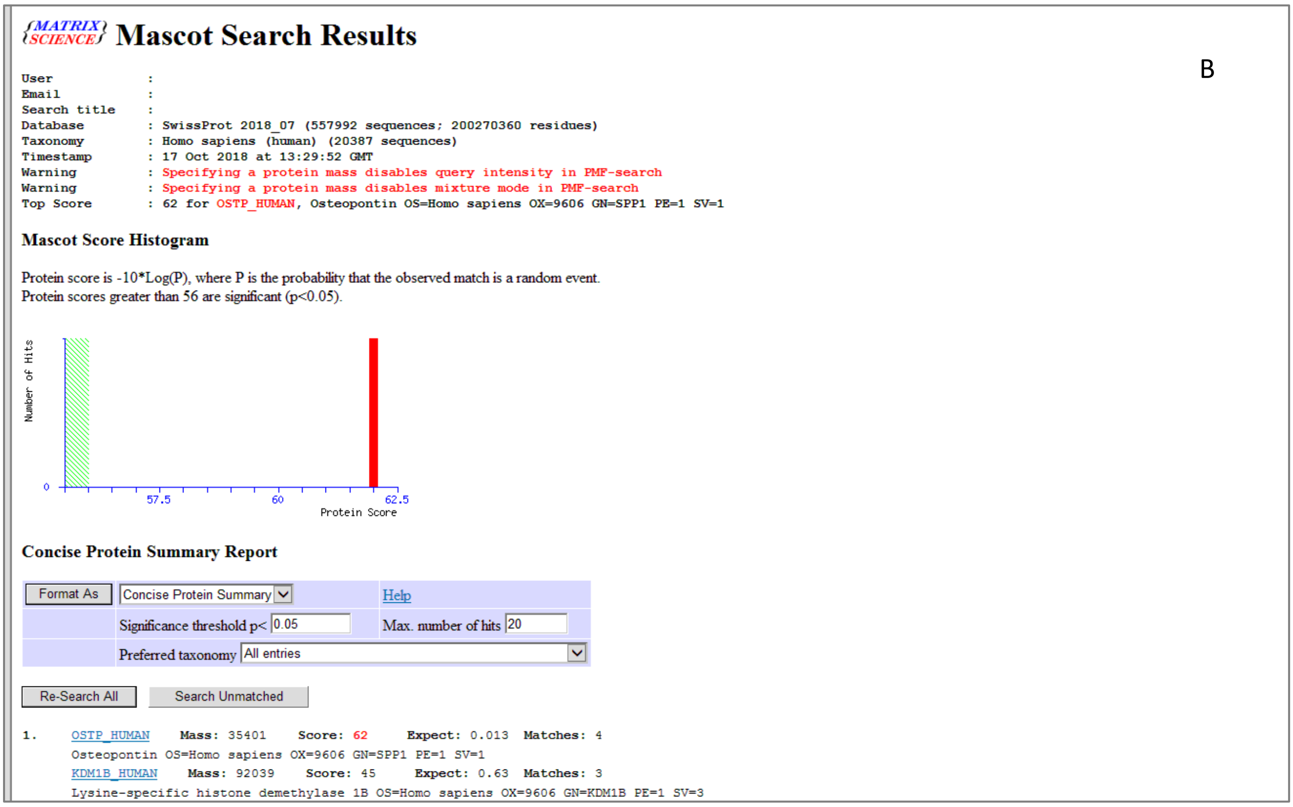

B

Mascot Search Results

```
User          :
Email         :
Search title  :
Database      : SwissProt 2018_07 (557992 sequences; 200270360 residues)
Taxonomy      : Homo sapiens (human) (20387 sequences)
Timestamp     : 17 Oct 2018 at 13:29:52 GMT
Warning       : Specifying a protein mass disables query intensity in PMF-search
Warning       : Specifying a protein mass disables mixture mode in PMF-search
Top Score     : 62 for OSTP_HUMAN, Osteopontin OS=Homo sapiens OX=9606 GN=SPP1 PE=1 SV=1
```

Mascot Score Histogram

Protein score is -10*Log(P), where P is the probability that the observed match is a random event.
Protein scores greater than 56 are significant (p<0.05).

Concise Protein Summary Report

Format As | Concise Protein Summary | Help
Significance threshold p< 0.05 | Max. number of hits 20
Preferred taxonomy All entries

Re-Search All | Search Unmatched

```
1.  OSTP_HUMAN   Mass: 35401   Score: 62   Expect: 0.013   Matches: 4
    Osteopontin OS=Homo sapiens OX=9606 GN=SPP1 PE=1 SV=1
    KDM1B_HUMAN   Mass: 92039   Score: 45   Expect: 0.63   Matches: 3
    Lysine-specific histone demethylase 1B OS=Homo sapiens OX=9606 GN=KDM1B PE=1 SV=3
```

S2 Fig. Biotools Mascot identification results for MS ions of trypsin digest from 4 µg/ml rhOPN reference samples. (A) $n_{trypsin}$: n_{rhOPN} = 1:25. (B) $n_{trypsin}$: n_{rhOPN} = 1:5
