# Supplementary material for: An antibody-free sample pretreatment method for osteopontin combined with MALDI-TOF MS/MS analysis
Source: PLoS One. 2019 Mar 7;14(3):e0213405. doi: 10.1371/journal.pone.0213405 (PMC6405093; doi:10.1371/journal.pone.0213405)
Supplement: S3 Fig — (A) ntrypsin: nrhOPN = 1:1. (B) ntrypsin: nrhOPN = 5:1 (PDF) [file pone.0213405.s007.pdf]

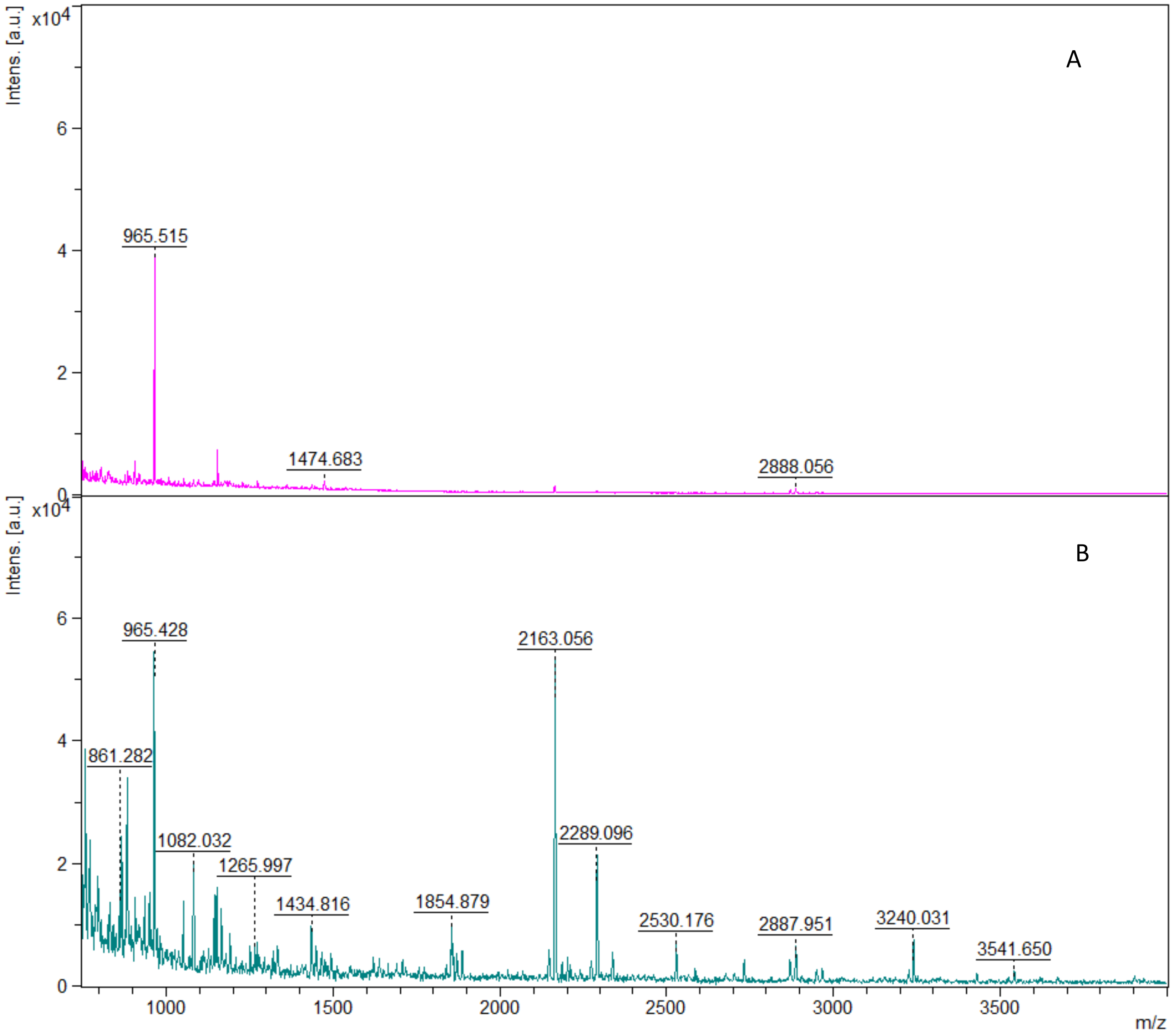

S3 Fig. MALDI-TOF MS of 200 ng/mL rhOPN trypsin digests with different molar ratio of trypsin to rhOPN. (A) $n_{trypsin}$: n_{rhOPN} = 1:1. (B) $n_{trypsin}$: n_{rhOPN} = 5:1
