# Supplementary material for: An antibody-free sample pretreatment method for osteopontin combined with MALDI-TOF MS/MS analysis
Source: PLoS One. 2019 Mar 7;14(3):e0213405. doi: 10.1371/journal.pone.0213405 (PMC6405093; doi:10.1371/journal.pone.0213405)
Supplement: S4 Fig — Left: rhOPN (200 μg/mL). Right: protein mixture (BSA 40 mg/mL, IgG 10 mg/mL, rhOPN 200 μg/mL). (PDF) [file pone.0213405.s008.pdf]

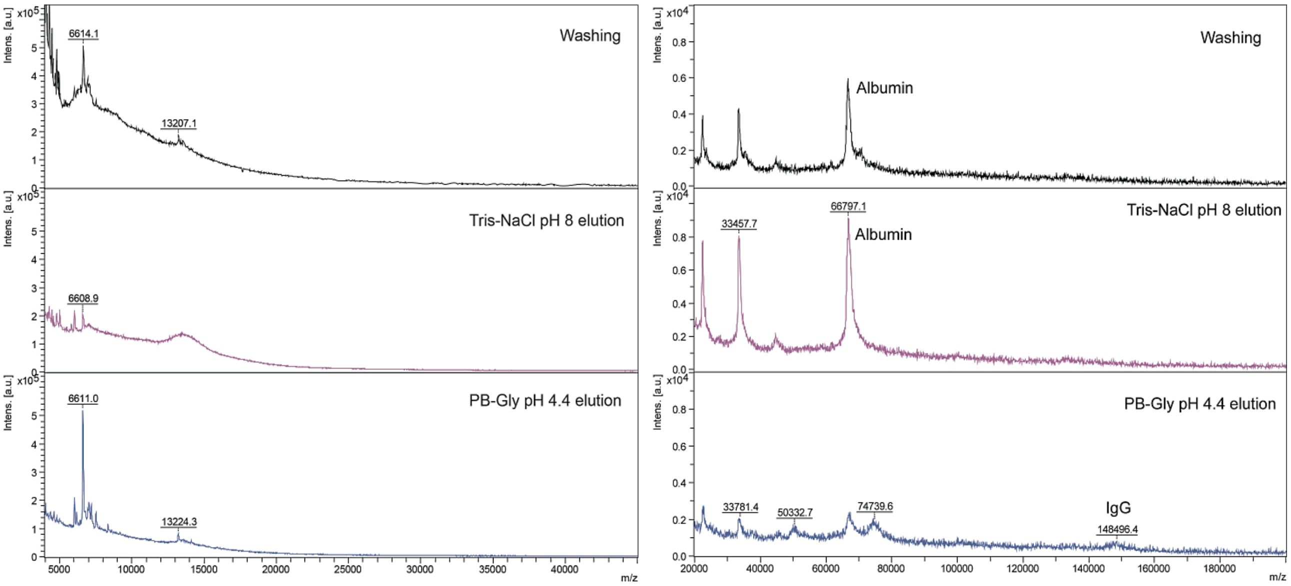

S4 Fig. MALDI-TOF MS of proteins in different extraction fractions using Protein A as affinity material. Left: rhOPN (200 µg/mL). Right: protein mixture (BSA 40 mg/mL, IgG 10 mg/mL, rhOPN 200 µg/mL).
