# Supplementary material for: An antibody-free sample pretreatment method for osteopontin combined with MALDI-TOF MS/MS analysis
Source: PLoS One. 2019 Mar 7;14(3):e0213405. doi: 10.1371/journal.pone.0213405 (PMC6405093; doi:10.1371/journal.pone.0213405)
Supplement: S5 Fig — (A) trypsin digests of Elution fraction 3 from 1 mg/mL rhOPN in human plasma sample. (B) trypsin digests of pure human plasma. (C) Comparison of the main peak m/z 1854.898 by superimposing spectrum (A) and (B). (PDF) [file pone.0213405.s009.pdf]

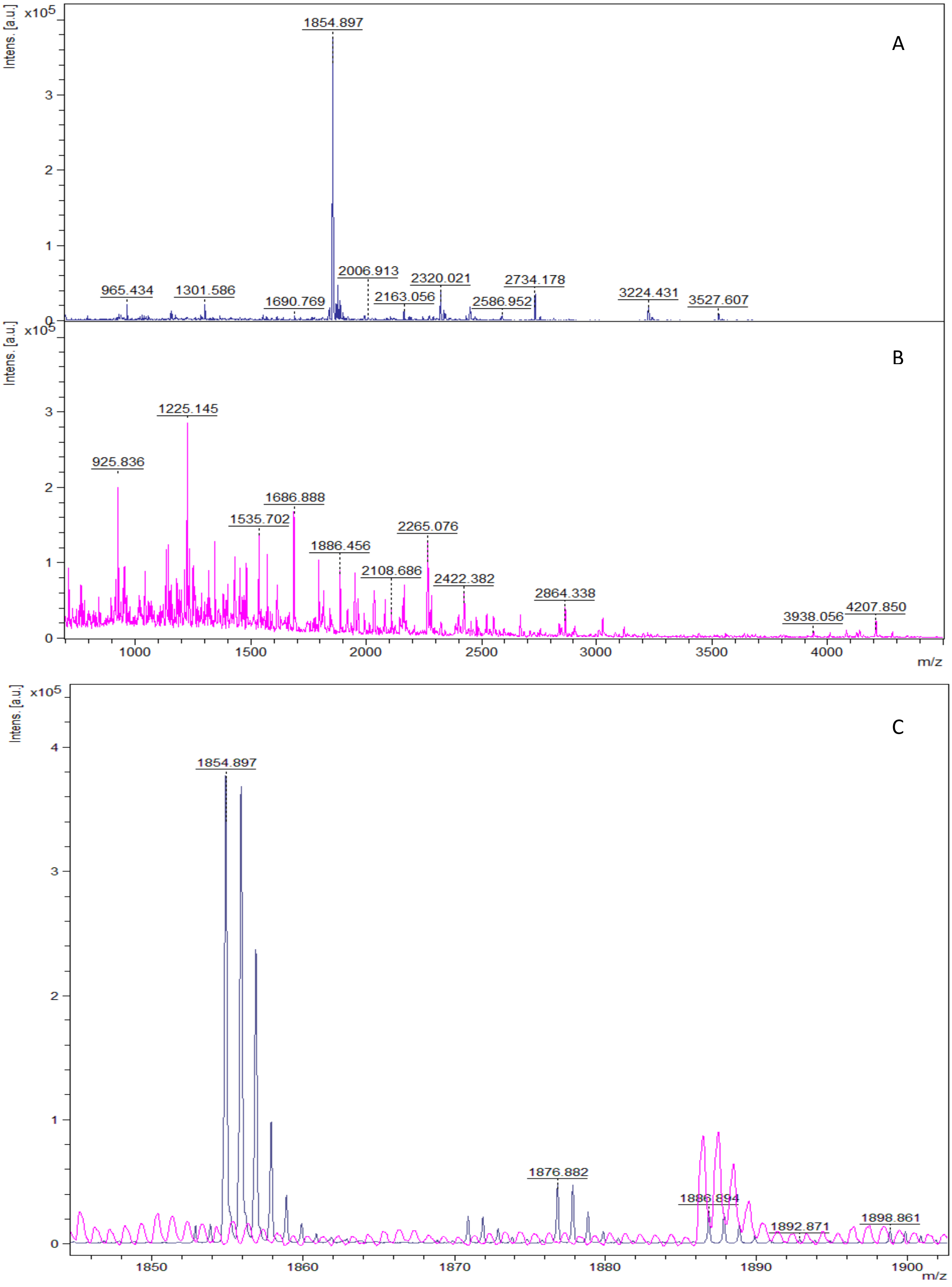

S5 Fig. MALDI-MS of trypsin digests of plasma samples. (A) trypsin digests of Elution fraction 3 from 1 mg/mL rhOPN in human plasma sample. (B) trypsin digests of pure human plasma. (C) Comparison of the main peak *m/z* 1854.898 by superimposing spectrum (A) and (B).
