# Supplementary material for: An antibody-free sample pretreatment method for osteopontin combined with MALDI-TOF MS/MS analysis
Source: PLoS One. 2019 Mar 7;14(3):e0213405. doi: 10.1371/journal.pone.0213405 (PMC6405093; doi:10.1371/journal.pone.0213405)
Supplement: S7 Fig — (A) Score for identification. (B) Identified MS/MS fragments of peak m/z 1854.898 and their corresponding sequences in human OPN. (PDF) [file pone.0213405.s011.pdf]

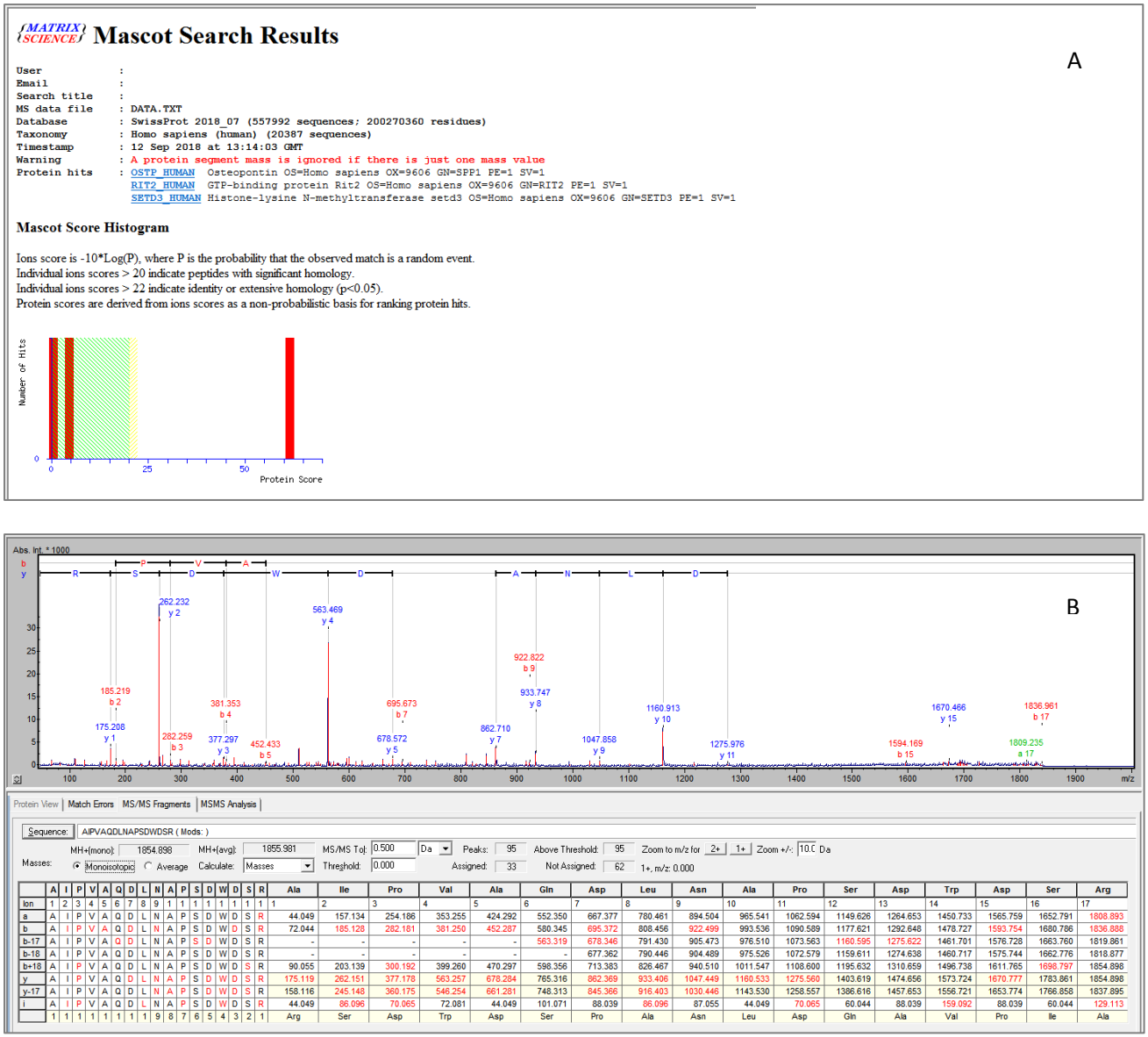

S7 Fig. Biotools Mascot identification results for MS/MS on the peak at m/z 1854.898 of trypsin digest from Elution fraction 3, extracted from rhOPN (100 µg/mL) in human plasma. (A) Score for identification. (B) Identified MS/MS fragments of peak m/z 1854.898 and their corresponding sequences in human OPN.
