# Supplementary material for: An antibody-free sample pretreatment method for osteopontin combined with MALDI-TOF MS/MS analysis
Source: PLoS One. 2019 Mar 7;14(3):e0213405. doi: 10.1371/journal.pone.0213405 (PMC6405093; doi:10.1371/journal.pone.0213405)
Supplement: S8 Fig — MALDI-TOF MS of 100 μg/mL rhOPN trypsin digests: (A) without dephosphorylation. (B) and (C) dephosphorylation before digestion. (D) and (E) dephosphorylation after digestion. (B) and (D) 0.25 unit phosphatase. (C) and (E) 1 unit phosphatase. (PDF) [file pone.0213405.s012.pdf]

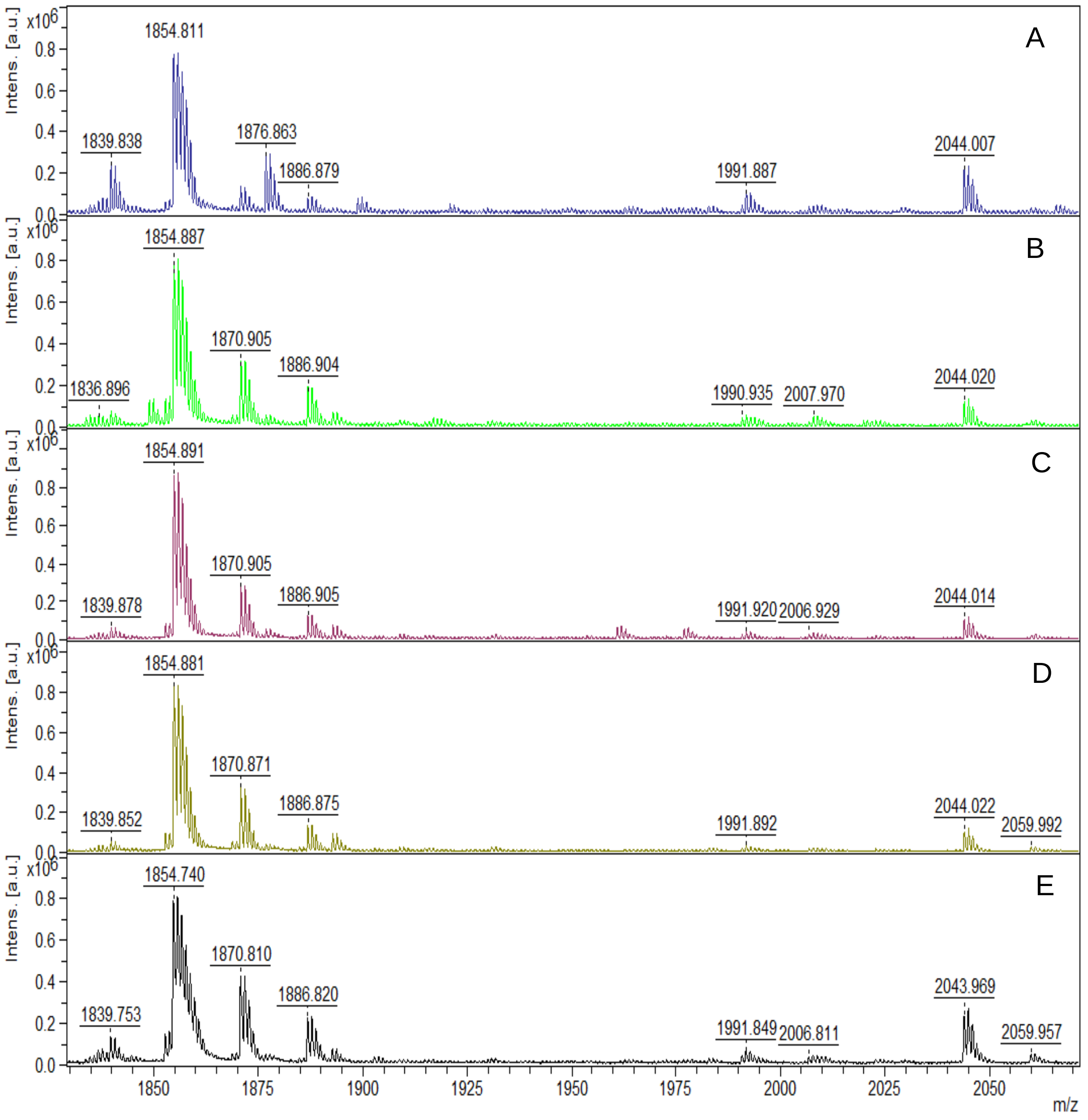

S8 Fig. MALDI-TOF MS of 100 µg/mL rhOPN trypsin digests: (A) without dephosphorylation. (B) and (C) dephosphorylation before digestion. (D) and (E) dephosphorylation after digestion. (B) and (D) 0.25 unit phosphatase. (C) and (E) 1 unit phosphatase.
