# Supplementary material for: An antibody-free sample pretreatment method for osteopontin combined with MALDI-TOF MS/MS analysis
Source: PLoS One. 2019 Mar 7;14(3):e0213405. doi: 10.1371/journal.pone.0213405 (PMC6405093; doi:10.1371/journal.pone.0213405)
Supplement: S9 Fig — (A) 10 mM Tris-HCl pH 8 + 0.1 M NaCl, (B) 10 mM Tris-HCl pH 8 + 0.2 M NaCl, (C) 10 mM Tris-HCl pH 8 + 0.3 M NaCl, (D) 10 mM Tris-HCl pH 8 + 0.4 M NaCl, (E) 10 mM Tris-HCl pH 8 + 0.5 M NaCl. (PDF) [file pone.0213405.s013.pdf]

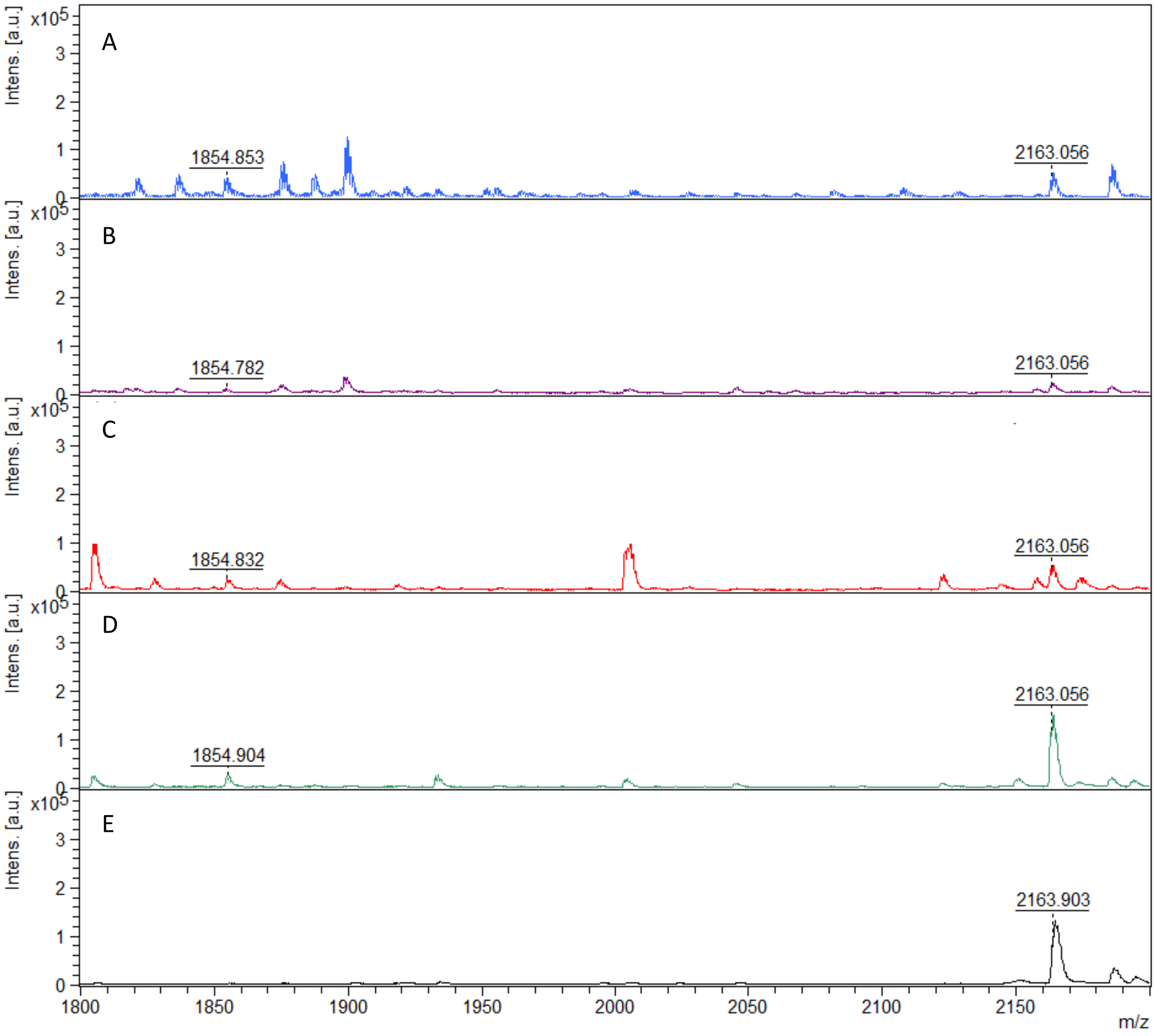

S9 Fig. MALDI-MS of trypsin digests from elution fractions with 20 µg/ml rhOPN in human plasma. (A) 10 mM Tris-HCl pH 8 + 0.1 M NaCl, (B) 10 mM Tris-HCl pH 8 + 0.2 M NaCl, (C) 10 mM Tris-HCl pH 8 + 0.3 M NaCl, (D) 10 mM Tris-HCl pH 8 + 0.4 M NaCl, (E) 10 mM Tris-HCl pH 8 + 0.5 M NaCl.
