# Supplementary material for: An antibody-free sample pretreatment method for osteopontin combined with MALDI-TOF MS/MS analysis
Source: PLoS One. 2019 Mar 7;14(3):e0213405. doi: 10.1371/journal.pone.0213405 (PMC6405093; doi:10.1371/journal.pone.0213405)
Supplement: S10 Fig — (A) Binding buffer 10 mM NaH2PO4 pH 4. (B) Binding buffer 10 mM NaH2PO4 pH 8. (C) Binding buffer 10 mM Tris-HCl pH 4. (D) Binding buffer 10 mM Tris-HCl pH 8. (PDF) [file pone.0213405.s014.pdf]

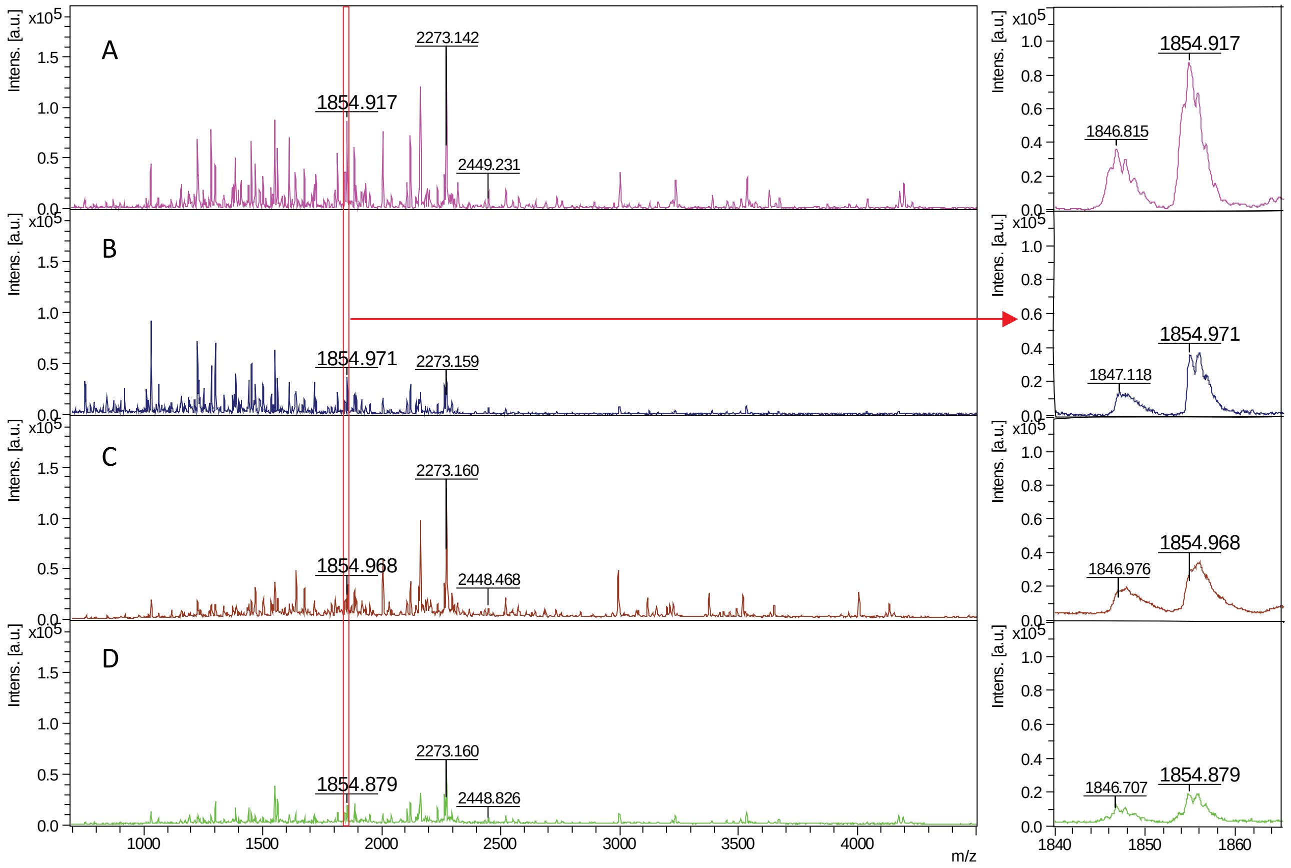

S10 Fig. MALDI-MS spectra for digested rhOPN, 20 µg/ml added in a human plasma sample, elution fraction 3. (A) Binding buffer 10 mM NaH_2PO_4 pH 4. (B) Binding buffer 10 mM NaH_2PO_4 pH 8. (C) Binding buffer 10 mM Tris-HCl pH 4. (D) Binding buffer 10 mM Tris-HCl pH 8.
