# Supplementary material for: An antibody-free sample pretreatment method for osteopontin combined with MALDI-TOF MS/MS analysis
Source: PLoS One. 2019 Mar 7;14(3):e0213405. doi: 10.1371/journal.pone.0213405 (PMC6405093; doi:10.1371/journal.pone.0213405)
Supplement: S11 Fig — (A) Eluted by PB-Gly-NaCl pH 4.4. (B) Eluted by PB-Gly-NaCl pH 8. (PDF) [file pone.0213405.s015.pdf]

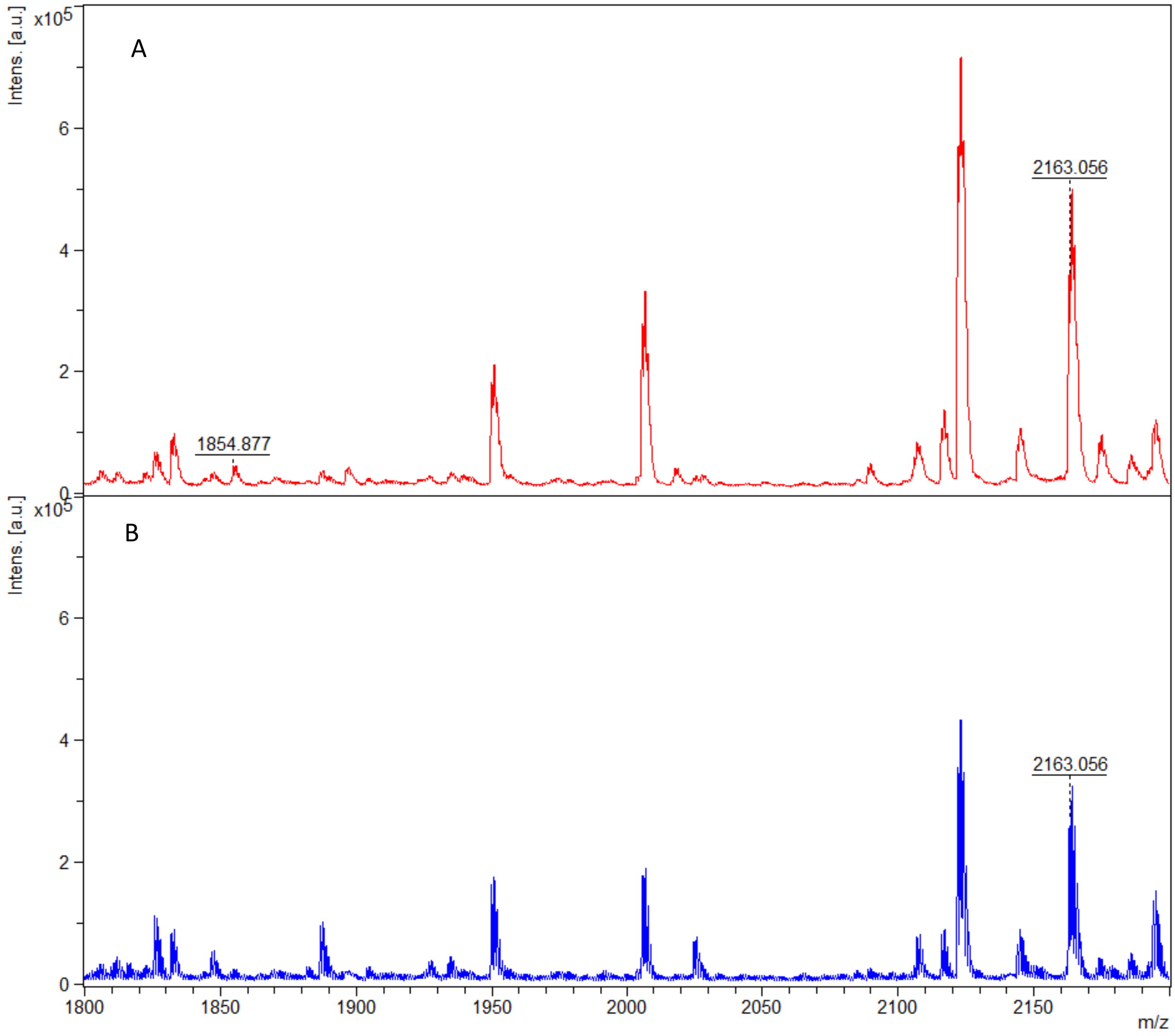

S11 Fig. MALDI-MS spectra for digested rhOPN, 1 µg/ml added in a human plasma sample, elution fraction 3. (A) Eluted by PB-Gly-NaCl pH 4.4. (B) Eluted by PB-Gly-NaCl pH 8.
