# Supplementary material for: An antibody-free sample pretreatment method for osteopontin combined with MALDI-TOF MS/MS analysis
Source: PLoS One. 2019 Mar 7;14(3):e0213405. doi: 10.1371/journal.pone.0213405 (PMC6405093; doi:10.1371/journal.pone.0213405)
Supplement: S12 Fig — (A) 2 μg/ml rhOPN, 0.5 μg trypsin. (B) 1 μg/ml rhOPN, 0.5 μg trypsin. (C) 2 μg/ml rhOPN, 0.25 μg trypsin. (D) 1 μg/ml rhOPN, 0.25 μg trypsin. (PDF) [file pone.0213405.s016.pdf]

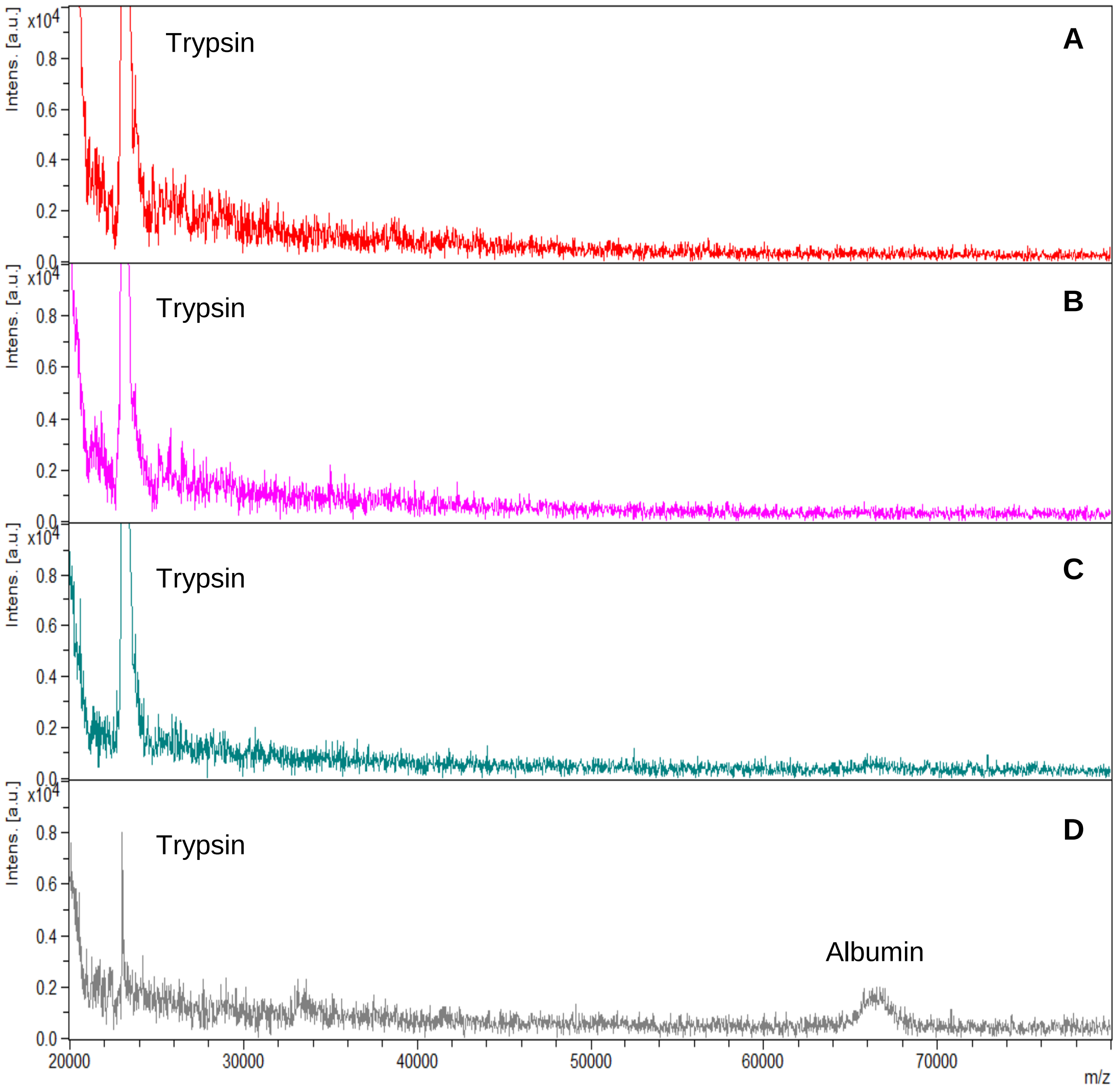

S12 Fig. MALDI-TOF MS of Elution fraction 3 from 1-2 µg/ml rhOPN in human plasma. (A) 2 µg/ml rhOPN, 0.5 µg trypsin. (B) 1 µg/ml rhOPN, 0.5 µg trypsin. (C) 2 µg/ml rhOPN, 0.25 µg trypsin. (D) 1 µg/ml rhOPN, 0.25 µg trypsin.
