# Supplementary material for: An antibody-free sample pretreatment method for osteopontin combined with MALDI-TOF MS/MS analysis
Source: PLoS One. 2019 Mar 7;14(3):e0213405. doi: 10.1371/journal.pone.0213405 (PMC6405093; doi:10.1371/journal.pone.0213405)
Supplement: S13 Fig — (A) rhOPN (2 μg/ml) in human plasma, DHB. (B) rhOPN (2 μg/ml) in human plasma, saturated HCCA. (C) rhOPN (1 μg/ml) in human plasma, 2.8 mg/mL HCCA. (D) rhOPN (1 μg/ml) in human plasma, saturated HCCA. (E) Identified MS/MS fragments of peak m/z 1854.898 from (D) and their corresponding sequences in human OPN. (PDF) [file pone.0213405.s017.pdf]

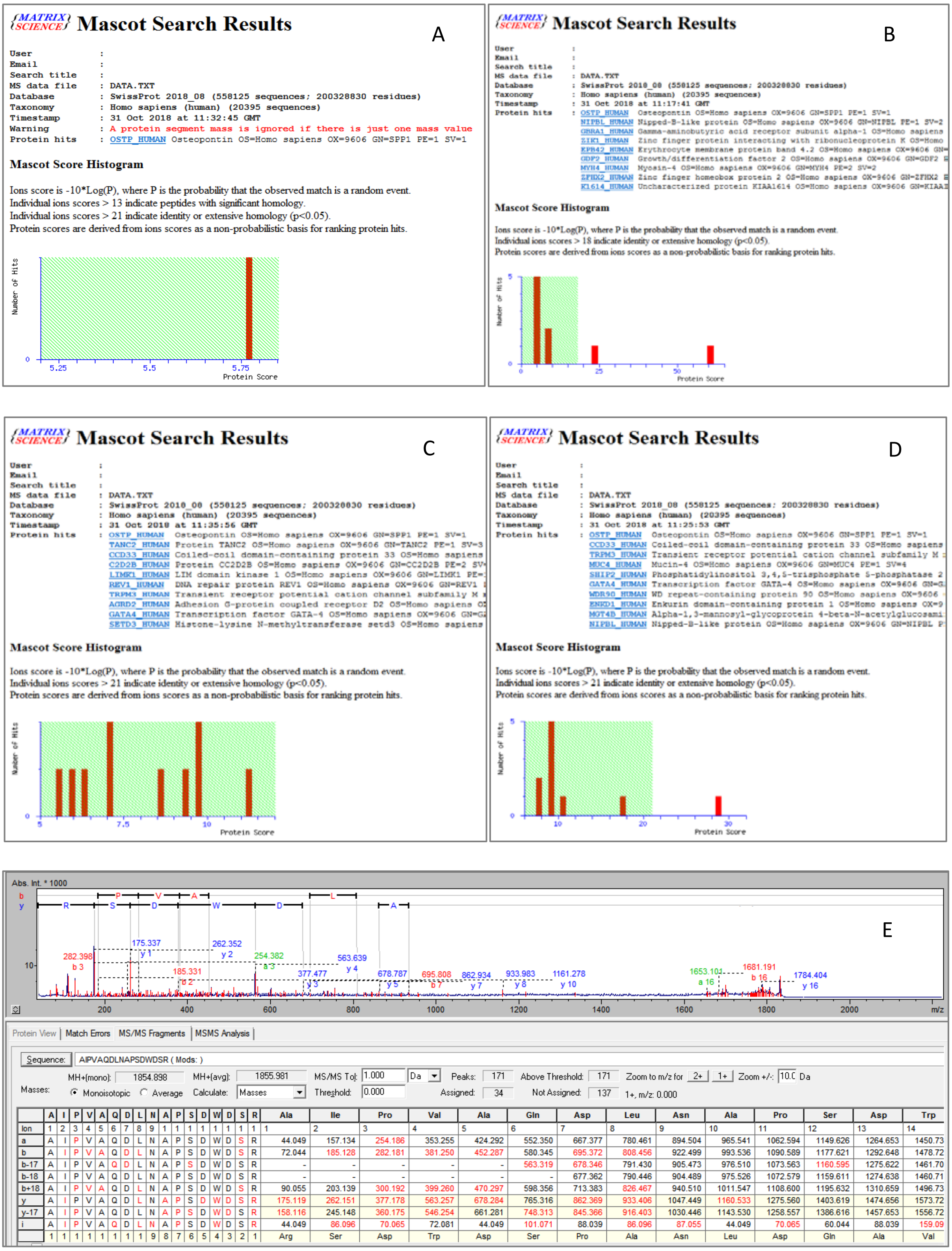

S13 Fig. Biotools Mascot identification results for MS/MS on the peak at m/z 1854.898 of trypsin digest from Elution fraction 3, extracted from plasma samples. (A) rhOPN (2 μg/ml) in human plasma, DHB. (B) rhOPN (2 μg/ml) in human plasma, saturated HCCA. (C) rhOPN (1 μg/ml) in human plasma, 2.8 mg/mL HCCA. (D) rhOPN (1 μg/ml) in human plasma, saturated HCCA. (E) Identified MS/MS fragments of peak m/z 1854.898 from (D) and their corresponding sequences in human OPN.
